# Role of Nrf2/HO-1 system in development, oxidative stress response and diseases: an evolutionarily conserved mechanism

**DOI:** 10.1007/s00018-016-2223-0

**Published:** 2016-04-21

**Authors:** Agnieszka Loboda, Milena Damulewicz, Elzbieta Pyza, Alicja Jozkowicz, Jozef Dulak

**Affiliations:** 1 Department of Medical Biotechnology, Faculty of Biochemistry, Biophysics and Biotechnology, Jagiellonian University, Gronostajowa 7, 30-387 Krakow, Poland; 2 Malopolska Centre of Biotechnology, Jagiellonian University, Krakow, Poland; 3 Department of Cell Biology and Imaging, Faculty of Biology and Earth Sciences, Jagiellonian University, Krakow, Poland

**Keywords:** Heme oxygenase-1 (HO-1), Kelch-like ECH-associated protein 1 (Keap1), Nuclear factor erythroid 2-related factor 2 (Nrf2), Oxidative stress, Reactive oxygen species (ROS)

## Abstract

The multifunctional regulator nuclear factor erythroid 2-related factor (Nrf2) is considered not only as a cytoprotective factor regulating the expression of genes coding for anti-oxidant, anti-inflammatory and detoxifying proteins, but it is also a powerful modulator of species longevity. The vertebrate Nrf2 belongs to Cap ‘n’ Collar (Cnc) bZIP family of transcription factors and shares a high homology with SKN-1 from *Caenorhabditis elegans or* CncC found in *Drosophila melanogaster.* The major characteristics of Nrf2 are to some extent mimicked by Nrf2-dependent genes and their proteins including heme oxygenase-1 (HO-1), which besides removing toxic heme, produces biliverdin, iron ions and carbon monoxide. HO-1 and their products exert beneficial effects through the protection against oxidative injury, regulation of apoptosis, modulation of inflammation as well as contribution to angiogenesis. On the other hand, the disturbances in the proper HO-1 level are associated with the pathogenesis of some age-dependent disorders, including neurodegeneration, cancer or macular degeneration. This review summarizes our knowledge about Nrf2 and HO-1 across different phyla suggesting their conservative role as stress-protective and anti-aging factors.

## Adaptive response to oxidative stress through Nrf2 and its target genes

Living organisms are frequently exposed to oxidative stress and toxic insults, like radiation, UV light, air pollution, toxins and others. Oxidative stress generated during such stressful conditions may damage DNA and proteins, and as a consequence the cellular processes are disturbed. Special cellular machinery should be activated to fight against increased reactive oxygen species (ROS) level to protect from oxidative injury. Single cells and organisms may adapt to harmful oxidative stress conditions, through stress-activated factors.

The nuclear factor erythroid 2-related factor 2 (Nrf2; encoded by *Nfe2l2* gene) is a transcription factor responsible for the regulation of cellular redox balance and protective antioxidant and phase II detoxification responses in mammals [1, 2]. The discovery of the antioxidant response element (ARE) have led to the conclusion that the battery of genes, including glutamate-cysteine ligase (GCL), thioredoxin reductase 1 (Txnrd1), NAD(P)H-quinone oxidoreductase 1 (NQO1) and heme oxygenase-1 (HMOX1) is regulated through Nrf2 binding to this consensus binding sequence [3]. This activates cascade of events which, in the end, affects oxidative status of the cells and provides robust protection against oxidative challenge.

**Fig. 1:**
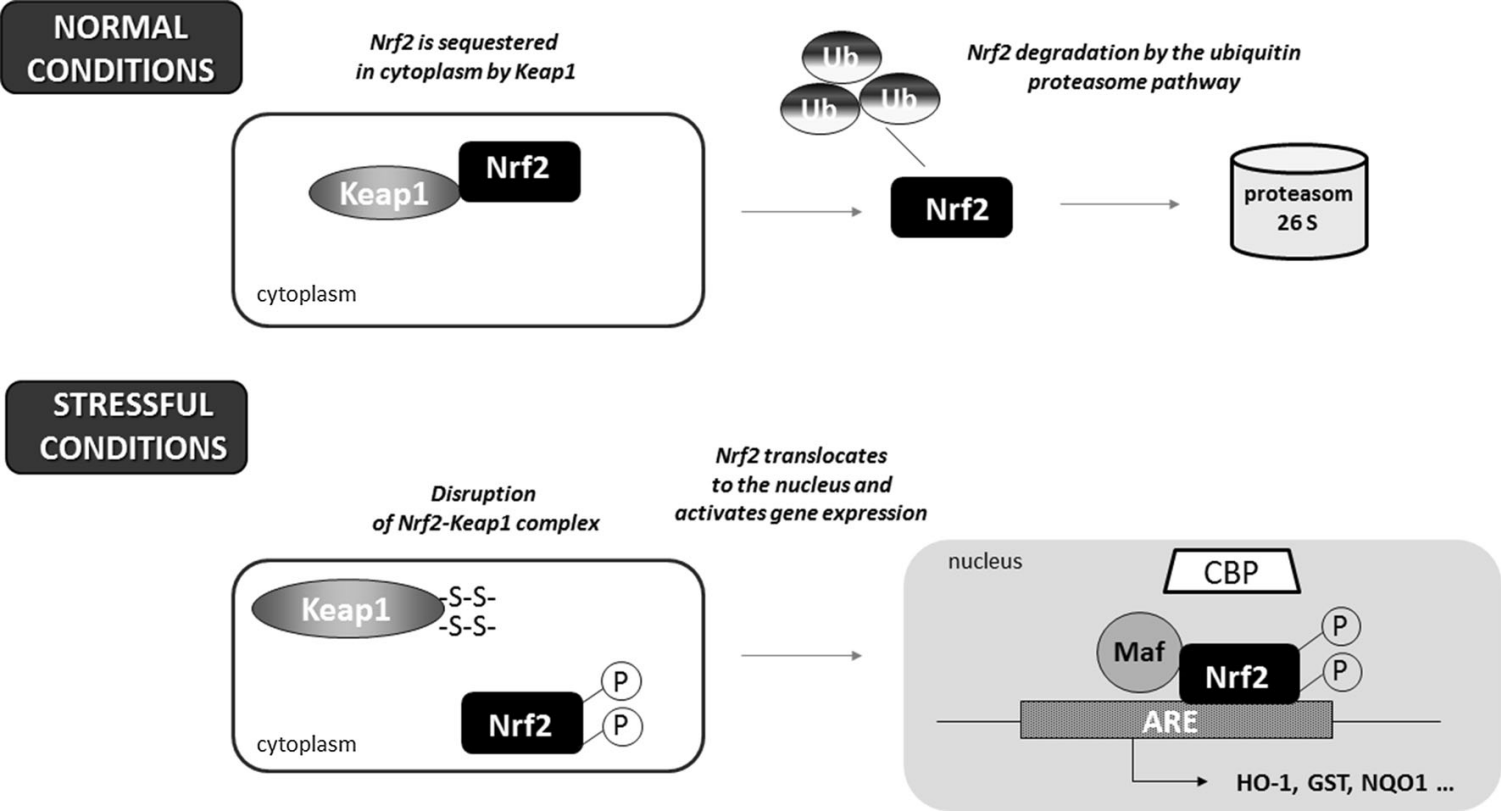
Schematic representation of the Nrf2-Keap1 pathway. Under normal conditions, Nrf2 is sequestered in cytoplasm by Keap1. In stressful conditions the modification of –SH groups in Keap1 or phosphorylation of Nrf2 facilitate the dissociation of Nrf2 from Keap1 as well as translocation of Nrf2 into the nucleus. After binding Maf proteins, Nrf2 activates antioxidant response element (ARE) and increases transcription of Nrf2-regulated genes (e.g., HO-1, GST, NQO-1)

**Fig. 2:**
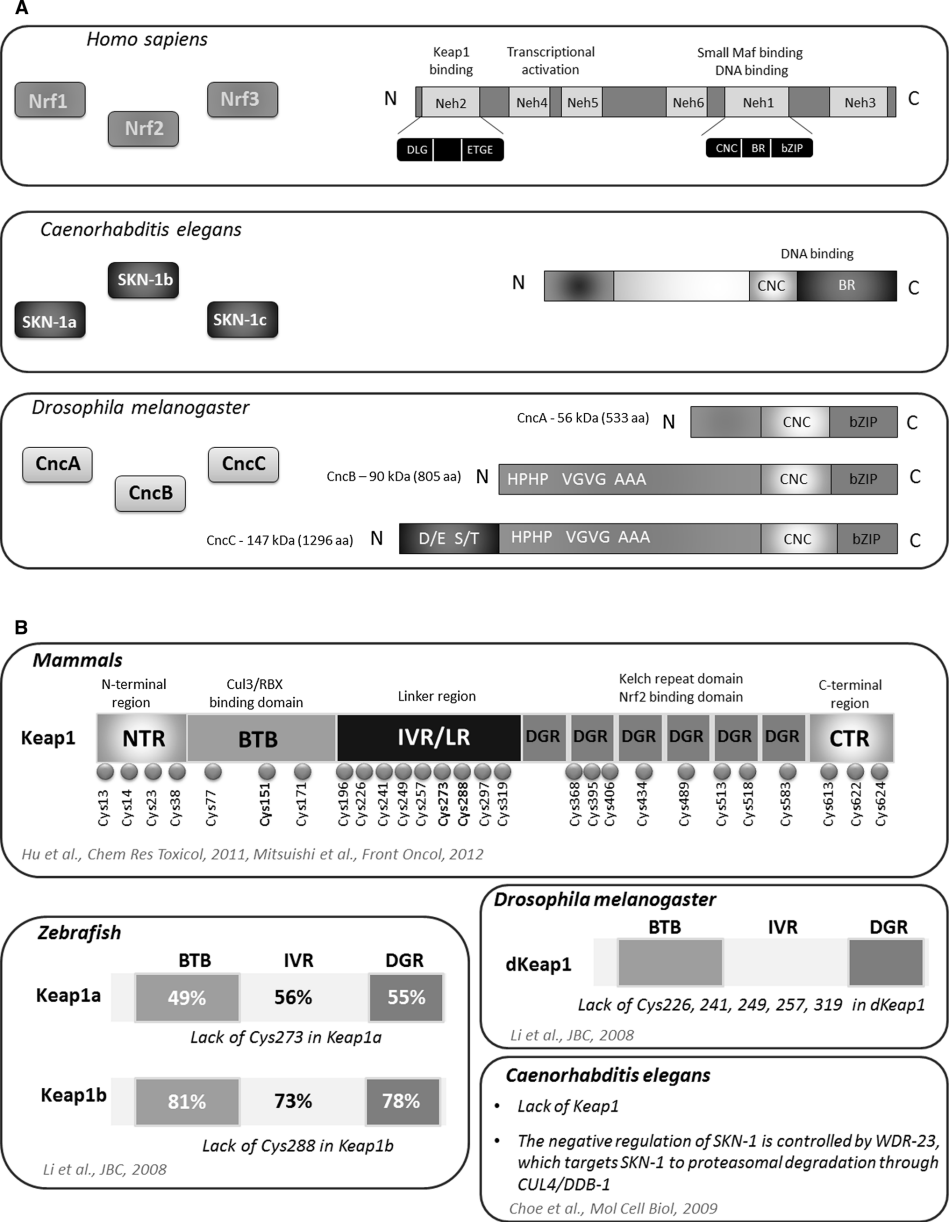
Complexity of CNC transcription factors and Keap1 regulator. CNC family of transcription factors share a high homology between *D. melanogaster, C. elegans* and *Homo sapiens.* From three Nrf factors found in vertebrates, the detailed domain structure of Nrf2 is shown. In *C. elegans*, three isoforms of SKN-1 factor possessing CNC domain responsible for DNA binding have been identified. In *D. melanogaster* CncA, CncB and CncC have distinct N-terminal end, but share very homologous C-terminus harboring the bZIP (**a**). Mammalian Keap1 consists of several domains which contain crucial cysteine residues. In zebrafish, two types of Keap1 with high homology to mammalian Keap1 are identified (similarity in amino acid sequences between zebrafish and mouse Keap1 shown in percentage). Keap1a and Keap1b, differ in the presence of essential Cys273 and Cys288, whereas dKeap1 in *Drosophila* lacks other cysteines but possess Cys273 and Cys288. In nematode, the function of negative regulator of SKN-1 is played by WDR-23 (**b**)

Nrf2 is a master eukaryotic redox-active factor and belongs to Cap ‘n’ Collar (Cnc)-bZIP (basic leucine zipper) family of transcription factors. Apart from Nrf2, also other NF-E2 p45-related factors 1 and 3 (Nrf1 and Nrf3) as well as transcriptional repressors Bach1 and Bach2 are the members of the family (reviewed in [4]). Nrf2 consists of six functional Neh domains (Neh1-Neh6), from which, the amino-terminal Neh2 domain controls binding Keap1—the inhibitor protein Kelch-like ECH-associated protein 1, that is responsible for the cytosolic sequestration of Nrf2 under physiological conditions (Fig. 2a). Keap1 is a cysteine-rich protein, known to be anchored to actin cytoskeleton [5], serving as an adaptor protein for the Cul3-dependent E3 ubiquitin ligase complex. Under normal conditions, Keap1 promotes ubiquitination and eventual degradation of Nrf2. This is a relatively rapid event, with Nrf2 exhibiting a short half-life of approximately 20 min [6]. On the other hand, under the stressful conditions, in which electrophiles and oxidants switch on Nrf2-dependent cellular defense mechanism, Nrf2 is released from Keap1 and translocates to the nucleus where it binds to conserved ARE sequence (reviewed in [7]). Keap1, as a thiol-rich protein, possesses at least 27 reactive cysteines that can be modified by electrophiles what leads to Keap1 inactivation and Nrf2 stabilization [8]. Nrf2 stabilization and increase in its half-life even to 200 min [9] allows nuclear translocation and activation of transcription of cytoprotective genes (Fig. 1). From abovementioned cysteines, two residues, Cys273 and Cys288, are crucial for Keap1 to control Nrf2 under both basal and stress conditions, whereas Cys151 is important for Keap1 activity predominantly in stressful conditions (reviewed in [10]).

As mentioned above, Neh2 domain identified in Nrf2 at the N-terminal end, is responsible for Keap1 binding. This interaction requires two key amino acid sequences within Neh2: ETGE and DLG (Fig. 2a). The other functional domains in Nrf2 play an important role in the regulation of transcriptional activity or its degradation. Neh4 and Neh5 domains are capable to interact with CREB-binding protein, CBP, enhancing the transcriptional activity of Nrf2. Neh6 is rich in serine residues, and this domain together with Neh2 plays a crucial role in Nrf2 degradation. The key Neh1 domain includes CNC-bZip motif responsible for DNA binding and dimerization with small Maf proteins. The next after Neh1, is the C-terminal Neh3 domain [11–14].

Similarly, in Keap1 protein-specific domains are identified. After the N-terminal region (NTR), the BTB domain (named after the *Drosophila* proteins Broad complex, Tramtrack, and Bric-a-brac, in which it was first identified), required for the formation of Keap1 homodimers and recruitment of Cullin-3 (Cul3) is present. The intervening region (IVR) also contributes to interaction with Cul3, whereas Nrf2 binding is controlled by the Kelch-repeat domain consisting of six repeats with double-glycine repeats (DGR)—key structural features of the Kelch domains (Fig. 2b) (reviewed in [10]).

## Evolutionary conservation of Cnc transcription factors

Nrf2-Keap1 system has been mostly studied in mammalian cells; however, a growing body of evidence suggest that similar pathways may play an important role in the protection against oxidative stress in other organisms. In 2002, Kobayashi et al. have identified and characterized the orthologues of Nrf2 and Keap1 in the zebrafish *Danio rerio* as the primary regulatory system of cytoprotective enzyme genes [15]. After treatment of the zebrafish larvae with *tert*-butylhydroquinone (tBHQ), the induction of cytoprotective and detoxifying genes like, *gstp1*, *nqo1* and *γgcsh* was observed, similarly to mammalian cells. Comparing Nrf2 of zebrafish and mouse, Kobayashi et al. found the sequence identity between the Neh1 and Neh2 domains of about 70 % [15]. In 2013, Williams et al. have demonstrated that the zebrafish expresses six *nrf* genes (*nfe2, nrf1a, nrf1b, nrf2a, nrf2b,* and *nrf3*). In this species, due to the whole genome duplication such multiplied copies (paralogues) that are co-orthologues of many single copy human genes are present [16]. Like the mammalian Nrf2, also in zebrafish this protein interacts with Keap1. Interestingly, two types of Keap1, Keap1a and Keap1b were identified by Li and coworkers [17]. It was shown that similarly to the mechanism observed in mammals, inhibition of Nrf2 by Keap1 is dependent on the interaction between Neh2 and Kelch domains as the specific mutation of the ETGE motif in Neh2 domain in zebrafish Nrf2 led to its escape from Keap1-mediated sequestration and nuclear localization [15, 17]. However, Keap1a and Keap1b lack a cysteine residue corresponding to mammalian Cys273 and Cys288, respectively [17]. Nevertheless, the presence of either Cys273 or Cys288 is sufficient for fish Keap1 molecules to exert full activity (Fig. 2b).

Similar factor to the mammalian Nrf2, responsible for oxidant signaling, was also identified in nematodes (Fig. 2). In 2003, An and Blackwell have shown that SKN-1 (skinhead-1) transcription factor is an orthologue of Nrf2 [18]. They and others have found that similarly to Nrf2, under normal conditions SKN-1 is sequestered in cytoplasm, but it translocates to the nucleus in response to stressful conditions, for example after exposure to H_2_O_2_. In the nucleus, it binds to DNA (to the consensus WWTRTCAT (W = A/T, R = G/A) sequence) as a monomer through a basic region (BR) [18]. Of note, *skn*-*1* gene encodes three splicing isoforms: SKN-1a, SKN-1b and SKN-1c, which share many similarities but they have also different expression patterns and functions (Fig. 2). SKN-1a plays a role in the endoplasmic reticulum stress response [19]. SKN-1b, expressed in a single pair of sensory neurons, controls lifespan in dietary restriction [20]. Finally, SKN-1c is accumulated in the intestine and it regulates expression of antioxidant and detoxification genes during stress response [18].

The negative regulation of SKN-1 is distinct from Nrf2 in mammals. A functional equivalent of mammalian Keap1, XREP-1 (WDR-23), was identified to control SKN-1 activity [21]. This regulation is based on the recruitment of SKN-1 to the CUL4/DDB1 (cullin 4/damaged DNA binding protein 1) ubiquitin ligase in nucleus leading to proteasomal degradation of this transcription factor and stopping its activity [22].

Additionally, in *Drosophila melanogastercnc* gene was identified and named based on its striking expression pattern in the anterior most labral segment (cap) and the mandibular segment (collar) of embryos [23]. The *cnc* gene encodes a bZIP transcription factor [23] and shares 60 % similarity over 160 aa with the p45 subunit of the NF-E2 transcription factor [24]. This homology includes 95 % identity in the 20 amino acid DNA-binding domain. Moreover, the DNA-binding domain of *cnc* is also shared with *skn*-*1* [23].

Due to alternative splicing Cnc has three isoforms: CncA, CncB and CncC which have distinct N-terminal end, but share very homologous C-terminus harboring the bZIP. Additional coding variants (longer isoforms) were identified and reported in Flybase [25], but their functions are not fully characterized. CncA and CncB are presumably maternal and they are detectable at syncytial and early cellular blastoderm stages, then they disappear until stage 14 [26]. CncA and CncC are required for germ cell viability and early oogenesis [27]. The *cncB* is expressed in an embryonic pattern that includes the labral, intercalary and mandibular segments. CncB protein is 272 amino acids longer than CncA isoform and it this part His-Pro, Ala, Val-Gly and Pro repeats are present (Fig. 2). This difference on the N-terminus is essential for repressive effect on Deformed (Dfd) response elements. CncC protein also possesses this 272 amino acid domain, and it has additional 491 amino acids, enriched in Ser-Thr and Asp-Glu motifs, at its N terminus that are unique to this isoform.

The molecular mechanism of stress response in insects is similar to that described in mammals. In unstressed conditions, CncC is retained in the cytoplasm by the actin-associated protein Keap1 [28]. Additionally, CncC activity is limited by dKeap1, which serves as an adaptor protein for Cul3-based ubiquitin ligase, targeting CncC for degradation [29]. The comparison of amino acid sequence homology between human Keap1 protein and *Drosophila* dKeap1 is presented in the recent review by Pitoniak and Bohmann showing conservation in BTB domain, linker domain and also in Kelch-repeat domain (see review [25]). Li and coworkers [17] have found that dKeap1 lacks Cys226, Cys241, Cys249, Cys257, Cys319 but possess the crucial Cys273 and Cys288, enabling dKeap1 to be fully active.

In normal conditions, AREs are blocked by small Maf (Maf-S) (musculo aponeurotic fibrosarcoma) protein [30, 31]. Maf-S does not have transcription activation domain, and after its binding to ARE, target gene expression is not activated. After stress exposure, electrophiles and ROS disrupt interaction between CncC and Keap1. CncC is not degraded and it is accumulated in the nucleus, where it forms heterodimers with Maf-S, binds to AREs and activates target genes transcription [32]. This mechanism is necessary for transcriptional responses to many xenobiotics, like chlorpromazine, caffeine or the pesticide malathion [29] and represents evolutionarily conserved mechanism of cell adaptations to stressful conditions.

In *Drosophila,* in contrast to mammals, where Nrf2 affects only phase II genes, CncC regulates the expression of genes involved in every phase of detoxification. It is important to remind that detoxification process may be divided into three phases. Phase I is realized by many enzymes, like cytochrome P450 monooxygenases, which decrease biological activity of xenobiotics. Phase II is a process of conjugation, when metabolites produced in the phase I are linked with carboxyl-, hydroxyl-, amino-, or sulfhydryl-groups. In this phase glutathione *S*-transferases (GSTs) or UDP-glucuronosyltransferases (UGTs) play the crucial role in the increase of hydrophilicity of xenobiotics and carboxylesterases. Phase III is a final modification and excretion, dependent on ATP-binding cassette (ABC) and other transmembrane transporters. Misra et al. have found that CncC causes changes in the expression of enzymes involved in all phases of detoxification, including 36 different P450, 17 GSTs, six UGTs and 55 different transporters [33]. It is worth to highlight that three P450 genes—*Cyp6g1, Cyp6g2 and Cyp12d1*—which are up-regulated by CncC, are responsible for pesticide resistance in insects [34, 35]. It was also shown that CncC/Keap1 pathway is constitutively active in insecticide-resistant *Drosophila* strains [32]. An additional protection against xenobiotics is CncC-dependent up-regulation of cuticle gene expression [33]. CncC also regulates many metabolic pathways which may be connected with detoxification. For example, CncC up-regulates *Zw* and *Pgd*, encoding glucose-6-phosphate dehydrogenase and phosphogluconate dehydrogenase, respectively. These enzymes are involved in pentose phosphate pathway and they are critical for NADPH production, which is essential for P450 and GST function [33]. Interestingly, Jones et al. showed that additional response to xenobiotics is provided by intestinal *Lactobacilli*, causing ROS-dependent activation of Cnc pathway [36].

In *C. elegans* oxidative stress causes accumulation of SKN-1 in intestinal nuclei and activation of gene expression from I, II and III detoxification phase. Moreover, SKN-1 induces the expression of C-type lectins, which are localized on the intestinal surface and are known as antimicrobial agents [37]. In addition, it has been suggested that SKN-1 maintains lipid homeostasis and it may be important for longevity [37]. This factor up-regulates lipid metabolism genes in starvation or dietary conditions, which induces fat mobilization. SKN-1 responds to elevated lipid levels by activation of gene transcription involved in β-oxidation, lipolysis, fatty acid desaturation, elongation, and transport [38].

In summary, CncC in *Drosophila*, SKN-1 in nematodes as well as vertebrate Nrf2 represent the same family of conserved factors having the unique 43-amino acid CNC domain located N-terminally to the DNA-binding domain (Fig. 2). These factors modulate the stress response in several scenarios, and therefore we discuss their potential role in both the normal and pathologic conditions.

## Role of Cnc transcription factors in development

**Fig. 3:**
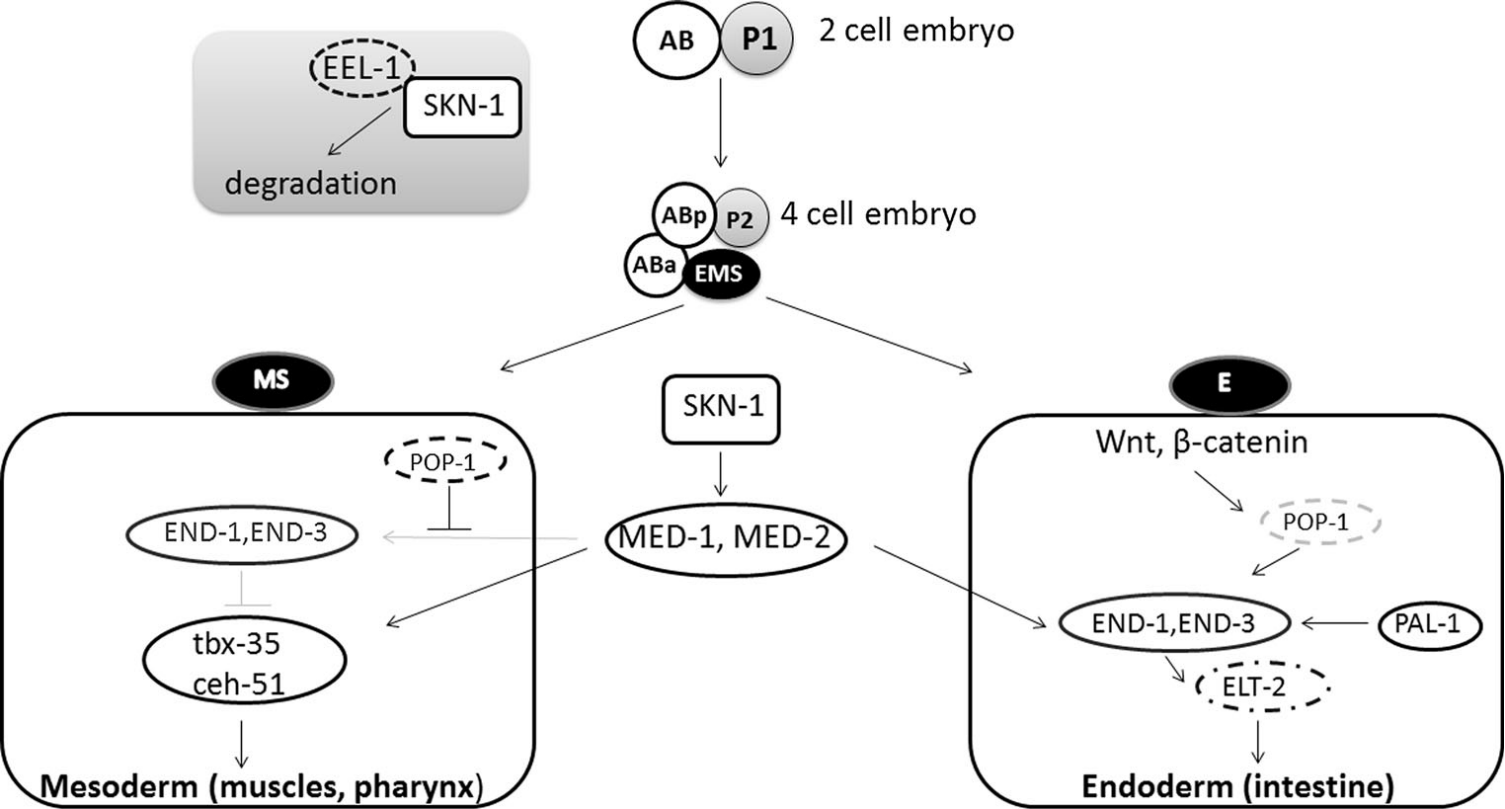
The role of SKN-1 in the development of *C. elegans*. In the early embryo *skn*-*1* mRNA is deposited in both cells: AB and P1, but the level of SKN-1 protein is higher in the posterior cell (P1). This asymmetry is regulated by EEL-1, which binds SKN-1 and targets it to degradation mainly in AB cell. After division AB cell forms Aba (anterior) and ABp (posterior) cells and P1 forms P2 and EMS cells. In this four-cell embryo, SKN-1 is concentrated mostly in EMS cell. In the next step, EMS divides into the mesodermal MS precursor, which develop into pharynx and muscles, and the endodermal E precursor, which generates intestine cells. SKN-1 increases level of the transcription factors MED-1 and MED-2, which in turn activates *end*-*1,3* transcription. In MS cell POP-1 protein inhibits END-1,3, whereas MED-1 and MED-2 activate transcription factors: tbx-35 and ceh-51, which are crucial for mesoderm development. In E cell POP-1 is modified on Wnt/β-catenin-dependent pathway, what activates END-1,3. END-1,3 cooperate with each other to increase the level of ELT-2, which starts intestine development

Transcription factors belonging to the CNC-bZIP family play a crucial role in an organism growth. SKN-1 is critical for development of the endoderm and mesoderm, especially it initiates the formation of the digestive system and other mesendodermal tissues in *C. elegans*. Interestingly, SKN-1 is asymmetrically distributed within the embryo (Fig. 3). In two-cell embryo *skn*-*1* mRNA is deposited in both cells [39], but protein is expressed at higher level in the posterior cell. This asymmetry is regulated by EEL-1, that is the Hect E3 ubiquitin ligase, targeting SKN-1 for degradation [40]. In four-cell embryo these differences in SKN-1 concentration are even more pronounced with high level of SKN-1 in the posterior daughter cells. SKN-1 determines the mesendodermal EMS precursor cell [41, 42]. EMS divides into the mesodermal MS precursor that develops into pharynx and muscles, and the E precursor which generates all 20 intestine cells. SKN-1 directly induces the expression of the transcription factors *med*-*1* and *med*-*2,* which activate expression of the GATA factors *end*-*1* and *end*-*3* in the E cell [43, 44]. In contrast, in the MS precursor maternally deposited POP-1 inhibits END-1, 3, and this results in blocking intestine development pathway. Moreover, in MS cells MED-1,2 activate T-box transcription factor tbx-35, that stimulates ceh-51, and then together they initiate a pathway for mesodermal specification [45, 46]. In the E precursor, POP-1 is modified through Wnt-dependent pathway, and this decreases its nuclear level and cooperation with β-catenin SYS-1 to activate END-1,3 [47, 48]. END-1,3 cooperate each other to activate *elt*-*2,* a crucial gene in intestine development [49–51]. After the eight-cell stage, EEL-1 downregulates SKN-1 and this protein is no longer detected in the early embryo [18, 40].

The role of CNC factors is *Drosophila* development is also well described. Cnc function starts during oogenesis, when particular maternal mRNAs are deposited in oocytes. These mRNAs are localized in a specific place of oocyte and after fertilization they are translated for proteins, which are necessary to form the anterio-posterior axis of embryo. Maternally deposited Cnc (A or C isoform) is required for proper microtubules positioning and affects anchoring of the nucleus to the anterior cortex of the oocyte [52].

*cncB* transcription is activated in blastoderm and continuous during embryogenesis in two parts of embryo: the anterior part called “cap” expression domain and in the mandibular segment that corresponds to the “collar” domain [23, 53]. The specific *cnc* expression pattern results from function of many genes, including *knot* (*kn*) [known also as *collier* (*col*)] [54, 55], *bicoid, torso*, *tailless* (*tll*)*, snail* (*sna*)*, spalt* (*sal*) and *giant* (*gt*) [56]. CncB controls development of cephalic segments: the anterior compartment of the mandibular segment and the labral segment [23]. In mandibular development CncB cooperates with homeotic *Deformed* (*Dfd*) gene. Tissues expressing CncB, but not Dfd, form hypopharynx. In mandibular cells and in some maxillary cells, CncB inhibits Dfd-activated gene transcription. Of note, tissues expressing both CncB and Dfd develop mandibular lobe-derived structures. Finally, tissues expressing Dfd only, (but not CncB) drive maxillary development [57]. Although the N-terminal of Cnc acts as a strong transcriptional domain, CncB function requires Maf-S protein, a homolog of the mammalian small Maf. Maf-S can potentially form heterodimers with all Cnc isoforms but, in contrast to the mammalian Maf protein, it is not able to form homodimers and to bind to DNA itself. CncB/Maf-S heterodimers bind a specific sequence (TGCTGAGTCAT) that is very similar to the mammalian Nrf/Maf binding site [31]. Other isoforms, CncA and CncC, also affect embryogenesis, but their specific functions are not well described yet [26].

**Fig. 4:**
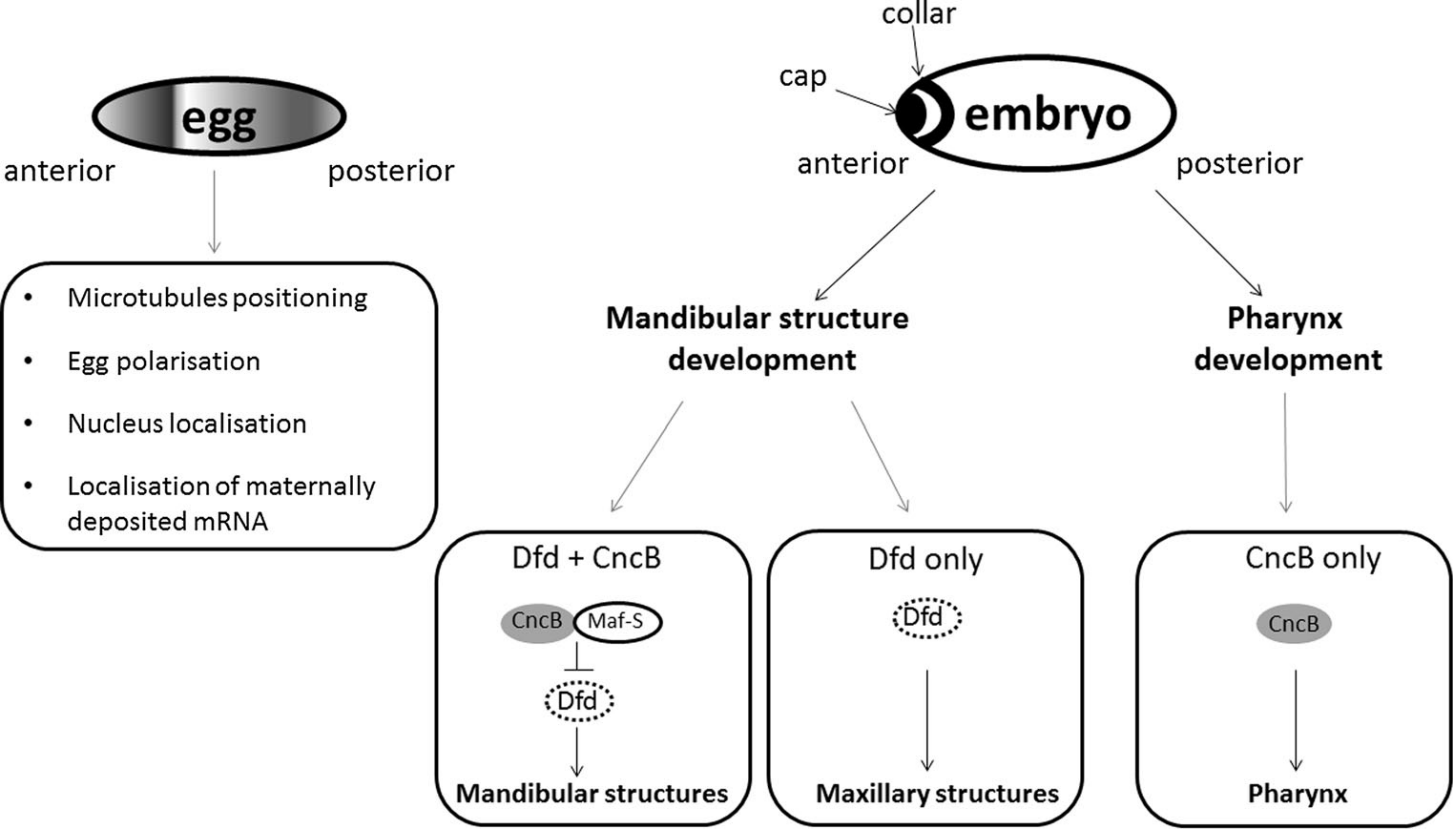
Cnc factors regulate development of *D. melanogaster.* Maternally deposited Cnc has important functions in egg polarization. In embryo Cnc is concentrated in the anterior part, in “cap” and “collar” domains. Cnc co-operates with Dfd in tissue development. In cells which express both Cnc and Dfd, Cnc forms heterodimers with MafS protein, and inhibits Dfd function. As the result, mandibular structures develop. Cells expressing only Dfd develop into maxillary structures. Finally, cell expressing Cnc only form pharynx structures

CncB is also crucial for development of the pharynx and it is a pharynx selector gene. In the labral and dorsal pharyngeal region Cnc maintains the specific pattern of segment polarity gene expression, *hedgehog* (*hh*) and *wingless* (*wg*) [31, 58]. Both proteins Hedgehog and Wingless are necessary to the pharynx development. The involvement of Cnc transcription factors in *Drosophila* development is schematically summarized in Fig. 4.

In *Drosophila*, CncC controls also metamorphosis through the regulation of ecdysone biosynthetic genes and ecdysone response genes. In this pathway, CncC requires *Drosophila* Keap1 (dKeap1) as a partner. Both, CncC and dKeap1 are localized predominantly in the nucleus. dKeap1 interacts with CncC and then binds to specific loci (ecdysone-regulated early puffs) on the polytene chromosomes and maintains open chromatin structure that facilitates transcription. The reduction of CncC or dKeap1 in the prothoracic gland, the organ producing ecdysone, leads to the reduction of ecdysone biosynthetic gene transcription, decrease in ecdysone level and delayed pupation. CncC may also interact with Ras signaling pathway in metamorphosis and transcriptional regulation. Phosphorylation of CncC in response to Ras and possibly other signals can regulate the distribution of CncC on chromatin, resulting in the regulation of development and metamorphosis [59, 60].

In *Drosophila*, Cnc is involved in the regulation of intestinal stem cells (ISC) maintenance. ISC are the only cells in the intestinal epithelium which proliferate and are responsible for regeneration of the midgut. This process needs to be precisely regulated because an excessive proliferation in the intestine can result in disruption of the intestinal epithelium by the accumulation of misdifferentiated ISC daughter cells, as it has been observed in stress conditions and aging. Hochmuth et al. showed that CncC-Keap1 regulate ISC proliferation rates by influencing the intracellular redox state. CncC is constitutively active in ISC of young, unstressed flies and maintain low ROS level leading to inhibition of ISC proliferation. In older flies, in oxidative stress conditions, CncC activity is repressed by Keap1, and increases proliferation rate [61].

In vertebrates, the regulation of the embryonic development by Nrf transcription factors was examined in several studies. It has been shown that deletion of Nrf1 in mice leads to anemia, defects in erythropoiesis and death [62]. In contrast to Nrf1, the inactivation of the *Nfe2l2* gene did not induce obvious defects in knockout mice, indicating that Nrf2 is dispensable for growth and development [63]. Similarly, also *Nrf3* knockout mice survive healthy [64]. Leung et al. [65] have observed that deficiency of both Nrf1 and Nrf2 results in early embryonic lethality of mice and this indicates a compensatory effect of Nrf1 in case of *Nfe2l2* knockout. Increased apoptosis has been observed in embryos lacking both transcription factors and fibroblasts isolated from Nrf1^−/−^ Nrf2^−/−^ mice exhibit increased intracellular ROS levels and enhanced sensitivity to oxidative stress.

## Heme oxygenase-1 as an Nrf2-dependent gene

The Keap1-Nrf2 pathway regulates the expression of numerous cytoprotective genes (Table 1) including antioxidant ones, genes encoding enzymes that participate in the synthesis and regeneration of glutathione, detoxifying molecules, proteins that regulate the expression of other transcription factors and growth factors and many more.

**Table 1:** Cytoprotective genes regulated by Nrf2 transcription factor

Gene	Abbreviation	Major function
Ferritin	Fn	Sequesters free iron
Glucose-6-phosphate dehydrogenase	G6PD	Provides NADPH to gluthathione reductase
Gluthahione peroxidase	GPx	Detoxifies peroxides and hydroperoxides
Gluthathione *S*-tranferases	GSTs	Catalyze the conjugation of the reduced form of glutathione (GSH) to xenobiotic substrates
Gluthathione reductase	GR	Catalyzes the reduction of glutathione disulfide (GSSG) to the sulfhydryl form of glutathione (GSH)
γ-Glutamylcysteine ligase	GCL	Catalyzes the rate limiting step in the cellular glutathione (GSH) biosynthesis pathway
Heme oxygenase-1	HO-1	Degrades heme and generates the antioxidant molecules, biliverdin and CO
NAD(P)H:quinone dehydrogenase 1	NQO1	FAD-binding protein, reduces quinones to hydroquinones
Sulfotransferases	SULFs	Catalyze sulfation of many xenobiotics
Superoxide dismutase	SOD	Catalyzes the dismutation of the superoxide radical (O_2_^−^) into molecular oxygen (O_2_) or hydrogen peroxide (H_2_O_2_)
Thioredoxin reductase	TR	Reduces thioredoxin
UDP-glucose dehydrogenase	UGDH	Converts UDP-glucose to UDP-glucuronate

One of the genes regulated through Nrf2 is heme oxygenase-1 (HO-1, *HMOX1,* EC 1.14.99.3) [66]. HO-1 is one of two distinct HO isoforms found in mammals. It serves as an inducible 32-kDa protein, highly upregulated by a number of stimuli like heme, nitric oxide, heavy metals, growth factor, cytokines, modified lipids and others. In contrast, HO-2 isoform is expressed constitutively (reviewed in [67]).

**Fig. 5:**
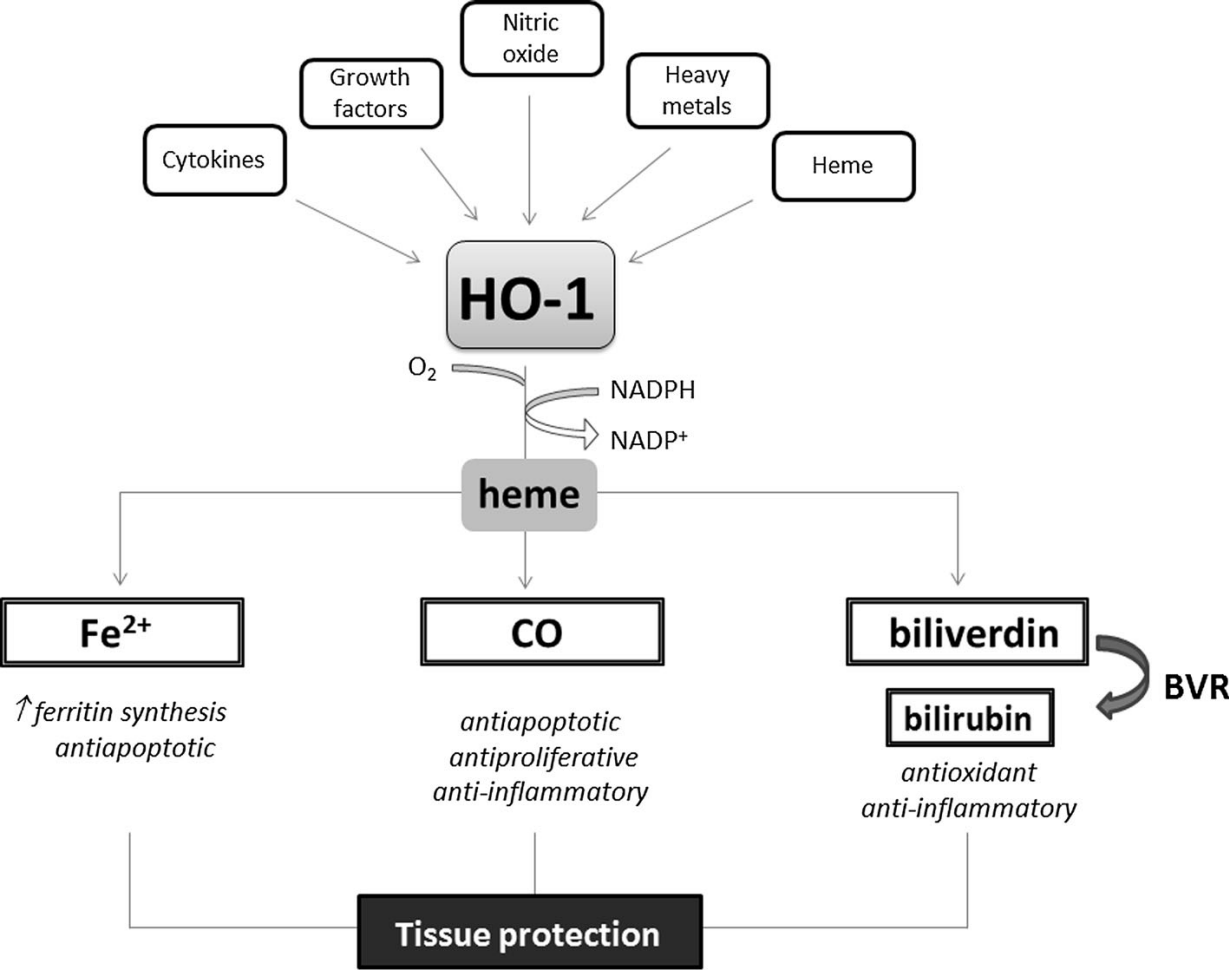
Heme oxygenase-1 pathway. HO-1, and inducible enzyme, cooperating with NADPH cytochrome P450 degrades heme to produce three bioactive products: iron ions, carbon monoxide and biliverdin, which is rapidly converted to bilirubin, through the action of biliverdin reductase (BVR). Tissue protection is exerted by the activity of all products and their functioning as a pro-angiogenic, antioxidant or anti-inflammatory factors

This cytoprotective enzyme catalyzes the rate-limiting step in heme degradation, leading to the generation of equimolar amounts of iron ions, biliverdin and CO (Fig. 5). Biliverdin together with bilirubin, formed thanks to the action of biliverdin reductase (BVR), are potent antioxidants, but also the other products of HO-1 activity regulate important biological processes including inflammation, apoptosis, cell proliferation, fibrosis, and angiogenesis. Iron is known to facilitate the generation of reactive oxygen species through Fenton reaction and might be possible toxic product of enzyme activity; however, already in 1992, Balla et al. [68] have shown that induction of HO-1 in endothelial cells exposed to H_2_O_2_-induced oxidative stress is accompanied with induction of ferritin, a protective enzyme sequestering iron ions. Such association between HO-1 and ferritin has been demonstrated also in vivo, e.g., in the inflammatory glomeruli in human lupus nephritis [69]. Both enzymes were expressed with similar pattern in the glomeruli and what is more, the transcript of ferritin heavy chain was decreased when HO-1 was knocked down. On the other hand, the stimulation of HO-1 was able to exert only partial protective effect against oxidative stress in cells lacking ferritin heavy chain, indicating cooperation of both enzymes [69]. It seems that CO is a major product of HO-1 activity playing a protective role both in physiology and in pathological conditions (for review see [70, 71]).

The importance of HO-1 has been underlined by the studies on HO-1 knockout animals. Poss and Tonegawa [72, 73] as well as Yet et al. [74] have created different strains of HO-1-null mice. In 1999, Yachie et al., reported the first known human case of HO-1 deficiency in 6-year-old boy with severe growth retardation [75]. Another case of human HO-1 deficiency was described 12 years later by Radhakrishnan et al. [76]. The results from those human cases of HO-1 deficiency generally confirmed animal studies. The major symptoms observed in HO-1-deficiency included enhanced systemic inflammation, abnormalities of the coagulation/fibrinolysis system, developmental failure, iron-deficiency anemia, intravascular hemolysis with fragmented erythrocytes, nephropathy, and vascular endothelial injury [75]. Almost the only one difference observed in human and mice deficiency was the asplenia in human case, whereas splenomegaly was noticed in the young knockout mice. Thus, one can sum up that the inadequate HO-1 level may result in a broad spectrum of severe side effects, which could affect many organs (reviewed in [67]). This may also explain why only two cases of human HO-1 deficiency have been described till now.

On the other hand, basal and induced expression of HO-1 can vary in the human population because of the highly polymorphic (GT)_*n*_ fragment in the promoter, which may significantly modulate cytoprotective, anti-inflammatory and pro-angiogenic functions of HO-1 [77]. The distribution of alleles with different (GT)_*n*_ lengths is bimodal within the human population, with the most frequent variants in Caucasians containing 23 and 30 repeats. The results from many studies have clearly shown that longer (GT)_*n*_ sequences are associated with diminished HO-1 expression and lower resistance to oxidative stress. Short (<25 GT) repeats are associated with higher HO-1 basal expression and stronger up-regulation after stimulation than longer repeats. The association between HO-1 polymorphism and occurrence/progression of some diseases, mostly cardiovascular disease risk has been suggested (reviewed in [67]). The recent study by Pechlaner et al. [78] confirmed previously reported relations and showed that subjects with ≥32 tandem repeats on both HO-1 alleles display substantially increased cardiovascular disease risk and enhanced atherosclerosis progression. However, human genome epidemiology (HuGE) meta-analysis of HO-1 polymorphism and susceptibility to coronary artery disease (CAD) from 11 studies, involving 10,170 patients with CAD and 6868 controls, performed in 2014 by Qiao et al. [79] revealed no significantly decreased risk of CAD in patients with the SS genotype of the HO-1 (GT)_*n*_ repeat length polymorphism compared with those with the LL + SL genotype. However, the results from the subgroup analyses showed that the (GT)_*n*_ SS genotype or S allele were associated with decreased CAD risks in Asian population and in studies with age- and sex-matched subgroups. Qiao et al. underlined that the lack of association between HO-1 polymorphism and CAD in Caucasian population found in their analysis may be at least partially caused by some limitations of the studies performed, and stressed the need of proper age and sex matching [79].

Besides cardiovascular disorders, the presence of long (GT)_*n*_ repeats has been suggested to be a prognostic marker for several cancer, but overall the results are complex. In oral squamous cell carcinoma [80], gastric adenocarcinoma [81] and lung adenocarcinoma [82] the lack of short allele corresponds with a higher incidence of cancer. On the other hand, in pancreatic cancer patients, an increasing risk was evident between LL, SL, and SS genotype, with the latter genotype displaying the most aggressive tumor biology [83]. Also in melanoma patients the homozygous short allele with <25 (GT)_*n*_ repeats (S/S) has been found more frequently in the melanoma group compared to the healthy control population and this genotype characterized tumors with deeper Breslow thickness compared to L-allele carriers [84]. However, similarly to cardiovascular disorders, the recent meta-analysis has shown lack of the association between HO-1 (GT)_*n*_ repeat polymorphism and cancer susceptibility. Zhang et al. [85] analyzed 10 studies involving 2367 cases and 2870 controls and found that overall cancer risk was not ascribed to the length of (GT)_*n*_ sequence; however, some sub-group analysis revealed higher risk of squamous cell carcinoma in persons carrying longer (GT)_*n*_ repeats in the HO-1 gene promoter. Nevertheless, in recent study, Wu et al. have found that participants with the SS genotype had an increased risk of lung squamous cell carcinoma versus those with L/S or L/L genotype. Moreover, in S/S genotype, higher incidence of Bowen’s disease and invasive skin cancer has been observed, while the participants with the S-allele had a reduced risk of lung adenocarcinoma versus those with L/L genotype [86].

Interestingly, the length of (GT)_*n*_ repeats in the HO-1 promoter did not influence the onset of neurodegenerative diseases such as Alzheimer’s disease (AD) and Parkinson’s disease (PD) [87, 88]. However, Infante et al. have shown that a −413 A/T single-nucleotide polymorphism (SNP) in HO-1, together with glycogen synthase kinase-3β (GSK3β) (−157, rs6438552) TT genotype had four times higher risk of developing PD than subjects without these genotypes [89].

## Role of HO-1 in embryogenesis and pregnancy

It is well established that HO-1-deficiency results in embryonic death and some studies evaluated the role of HO-1 in embryonic survival. HO-1 is expressed in the placenta and in the pregnant uterus already on embryonic day 6.5 (E6.5) [90]. Placenta of E14.5 embryos expresses very high levels of HO-1 mRNA, protein and activity [90]. Similarly, rat placenta expresses high HO-1 at day 15 of pregnancy [91]. Also in humans HO-1 is expressed in trophoblast and is highly induced during pregnancy, being a factor supporting pregnancy and decreasing abortions. Moreover, reduced HO-1 level was shown to be associated with miscarriages and pregnancy complication including pre-eclampsia (reviewed in [92]). Interestingly, the mother of the Japanese HO-1-deficient patient, was found to be heterozygous for *Hmox1* and she had experienced two intrauterine fetal deaths [75]. Also experiments on mice lacking both or one HO-1 allele support the crucial role of HO-1 in placenta and in pregnancy. Zhao et al. observed that HO-1 deficiency resulted in fetal lethality and decreased litter sizes [93] and also our own group have noticed abnormally low birth rate of HO-1^−/−^ offspring, born from HO-1^+/−^ parents (unpublished data). Intrauterine abortions of HO-1^−/−^ embryos, observed regularly in HO-1^+/−^ cross-breedings, were reported to occur before E10.5 [93]. Additionally, the weight of placenta in HO-1^+/−^ mice was reduced due to the apoptosis in the spongiotrophoblast layer. On the other hand, HO-1 overexpression was shown to improve pregnancy outcome in mice [94]. The detailed mechanisms responsible for HO-1 contribution to ovulation and fertilization, implantation and placentation is elegantly summarized in the recent review by Zenclussen [92]. In vivo experiments in rats showed the regulation of insulin-like growth factor (IGF) binding protein-1 by placental or trophoblastic HO-1 [95]. Noteworthy, IGF was reported to enhance the blastocyst formation, increase the number of blastomeres in cultured murine embryos, and to facilitate the establishment of stem cell lines [96]. HO-1 may also directly affect differentiation of pluripotent stem cells. Lin et al. generated induced pluripotent stem cells (iPSc) from embryonic fibroblasts, isolated from HO-1^+/+^, HO-1^+/−^, and HO-1^−/−^ mice, using Oct3/4, Sox2, Klf4 and c-Myc reprogramming factors [97]. After prolonged cell culture, the reduced expression of pluripotency markers in HO-1^−/−^ cells, increased sensitivity to oxidant-induced cell death and augmented differentiation were detected [97].

## Heme oxygenase system in prokaryotic and eukaryotic species

As it was described above, Nrf2 transcription factor shares high homology with their counterparts in *Drosophila*, nematodes or zebrafish, but importantly, they are not present in plants and fungi. In contrast, there is a high degree of evolutionary conservation of the heme-degrading enzymes among animal, plant, and fungal kingdoms. In vertebrates, the structural similarity and functional identity between HO enzymes is reported. Moreover, in bacteria, fungi, plants and flies, HO-1-like proteins or analogs have been identified.

Generally, in the mechanism of HO-catalyzed heme cleavage to α-isoform of biliverdin, CO and iron is universal among bacteria, fungi, plants, insects, and mammals. However, some differences might be present, when cofactor requirements or overall functional significance of heme metabolism between various organisms are discussed.

In *D. melanogaster,* HO-1 homolog (dHO) has been shown to degrade heme to biliverdin, CO and iron in the presence of reducing systems such as NADPH/cytochrome P450 reductase and sodium ascorbate, although the reaction rate is slower than that of mammalian HOs [98]. Interestingly, three isomers of biliverdin, biliverdin-IXα and two other isomers (IXβ and IXδ) are formed [98]. dHO has been shown to be not induced by stress stimuli; however, it is expressed in the brain in the circadian pattern [99, 100]. *ho* knockdown is lethal at larval stage, what suggests that dHO is essential for normal development [99]. The tissue-specific *ho* knockdown in the eye imaginal disc causes abnormalities in the eye morphology as well as of iron accumulation in the compound eye of adult flies. Surprisingly, heme is not accumulated in dHO-deficient tissues, due to decrease in the heme biosynthesis pathway, because the expression of delta-aminolevulinic acid synthase, the first enzyme of the heme-biosynthetic pathway is lowered in larvae as well [99].

In plants, the first HO homolog was identified in the red algae *Cyanidium caldarium* [101] and this enzyme was shown to generate biliverdin-IXα that is utilized for the phycobilin synthesis instead of the bilirubin production (generally, plants which lack the equivalent of mammalian BVR do not produce bilirubin) [102]. In *Arabidopsis thaliana*, *HY1* gene encodes a homolog of HO-1, and shows high sequence similarity to mammalian and cyanobacterial HOs as well as it possesses HO activity in vitro [103]. This homolog utilizes ferredoxin as the reducing partner instead of NADPH:cytochrome P-450 reductase [103], to generate biliverdin-IX as a precursor for the synthesis of light-harvesting pigments. Further analysis has underlined the existence of four members of HO family in *Arabidopsis thaliana* (HY1, HO2-4) [104]. Two subfamilies based on amino acid sequence alignments have been proposed: one subfamily includes HY1, HO3, and HO4 with the canonical HO active site, and another subfamily contains just HO2, which lacks the conserved histidine residue considered to be an important ligand for heme binding. The differences in the level of specific members were studied by Matsumoto et al. who showed that in *Arabidopsis* HO1 is the highly expressed, followed by lower level of HO2 and the lowest HO3 and HO4 [105].

In plants, the up-regulation of HO1 was observed in response to many agents, including light, UV, ROS, iron deprivation or cadmium (Cd), leading to the increased biosynthesis of phytochrome (phy) chromophore and thus being essential for proper photomorphogenesis. HO1 can also play a role in the protection against oxidative stress. For example, in soybean nodules, the treatment with 200 μM Cd caused tenfold induction of HO activity along with increased oxidative stress. Importantly, co-administration of 10 μM biliverdin completely prevented the effects caused by Cd, indicating a potent anti-oxidant activity of BV. On the other hand, the application of zinc protoporphyrin (ZnPPIX), an HO-1 inhibitor, significantly enhanced oxidative stress parameters [106].

Of note, heme oxygenases are present in prokaryotes as well (for review see [107]). Cyanobacterial HOs, e.g., isolated from *Synechocystis* sp. PCC6803, HO-1 and HO-2 are the best characterized bacterial HOs. In heme-utilizing bacterial pathogen *Corynebacterium diphtheriae*, HmuO enzyme has been shown to share 33 % sequence identity and 70 % homology to the human HO-1 [108]. On the other hand, HemO identified as the gene involved in the utilization of the host’s heme as an iron source and isolated from Gram-negative pathogen *Neisseriae meningitides* shows low sequence identity to other HOs [109].

The key functions in the catabolism of heme, regulation of oxidative stress, synthesis of phytobilins, bacterial pathogenesis and iron acquisition are only some of the numerous biological roles played by HOs in prokaryotes and eukaryotes.

## Cnc transcription factors may play a special role as pro-longevity and anti-aging factors

Nrf2 has been suggested as “a guardian of health span and gatekeeper of species longevity” [110]. Also in *D. melanogaster* CncC was demonstrated to be involved in aging processes, since older flies progressively lose the ability to CncC-targeted activation in response to stress. On the other hand, the expression of CncC target genes in unstressed conditions does not decrease in older flies [111].

Several mouse models of extended longevity have been utilized to check the role of Nrf2 in this process. The study performed in mice under calorie restriction, which is known to extend lifespan and delay many aspects of aging [112], showed that the lack of Nrf2 does not attenuate lifespan extension [113]. On the other hand, Nrf2 was prerequisite for cancer-preventive effects after the reduction of calories by 20–40 % [113]. However, experiments performed on *C. elegans*, indicate that the extended longevity of diet-restricted worms results from SKN-1 acting in special neurons, the ASIs. They signal to peripheral tissues to increase their metabolic activity [20]. The recent study by Smith-Vikos et al. identified the specific microRNAs that are critical for the dietary restriction-induced lifespan extension in *C. elegans*. miR-71 and miR-228 are induced by the calorie restriction and the regulation of these microRNA depends on SKN-1 [114]. Importantly, miR-71 has been shown to promote longevity because miR-71 loss-of-function mutants have significantly shorter lifespan than wild-type, and overexpressing miR-71 increases lifespan [115].

SKN-1 mutants have the shortened lifespan and oppositely, overexpression causes the extension of lifespan [18, 116]. Moreover, similarly to *Drosophila*, the expression level of SKN-1-regulated genes decreases with aging. SKN-1-mediated regulation of collagen expression which is necessary to extracellular matrix remodeling seems to be very important for aging and longevity [117]. This process is connected with the reduced insulin signaling in older animals, which normally regulates SKN-1 activity [118, 119]. SKN-1 is also involved in lifespan extensions in response to reduced activity of the mechanistic target of rapamycin (mTOR) signaling [120, 121].

Palikaras et al. showed that longevity depends on homeostasis between mitophagy (an autophagy type targeting mitochondria for degradation) and mitochondrial biogenesis in *C. elegans*. This process is regulated by SKN-1 by enhancing DCT-1 expression, a *C. elegans* homolog of mammalian BNIP3 and BNIP3L/NIX, key mediators of mitophagy [122, 123], for review see [124].

**Fig. 6:**
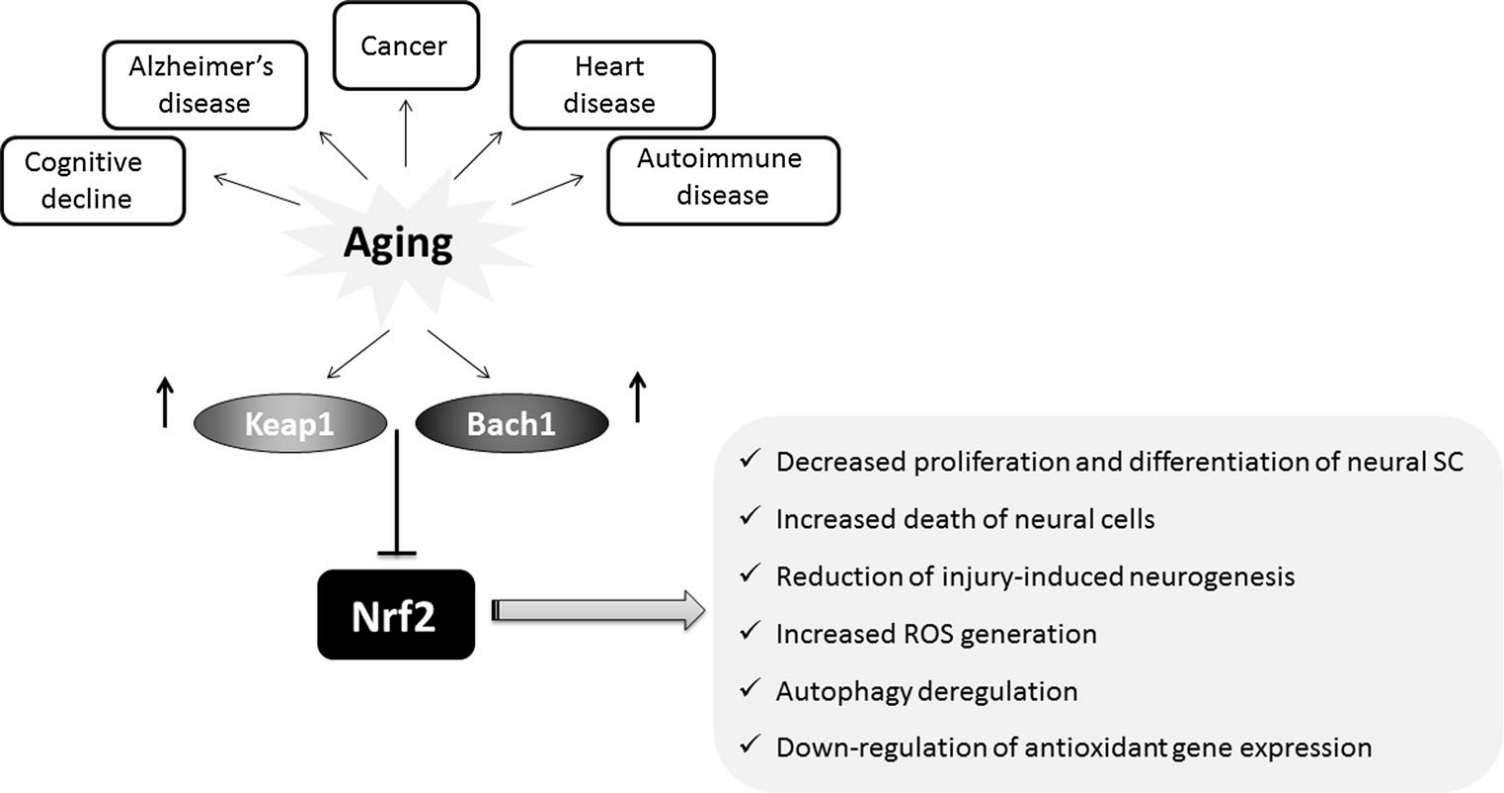
Nrf2 activity decreases with aging. Age-related changes in the Nrf2 regulatory system, including increase in Bach1 and Keap1 lead to the inhibition of Nrf2 activity and downstream effects

Interestingly, in the model of Keap1 inactivation in *D. melanogaster*, males showed the higher survival rate after paraquat (a free radical generator) exposure. Moreover, under standard culture conditions, male *keap1*^−/+^ heterozygote flies live significantly longer than their sibling controls [29]. In addition, in Ames dwarf mice and Little mice, the models of delayed aging, showing significant increases in lifespan (50 and 25 %, respectively), the enhanced activity of detoxification enzymes and antioxidant proteins including GST (Nrf2-target gene) in comparison with their control counterparts was observed [125]. The recent study performed in ten rodent species, ranging in the maximum lifespan potential (MLSP) between 4 and 31 years, has shown that in the long-lived naked mole rats there is a constitutively high level of Nrf2 in comparison to other rodent species with shorter lifespan [126]. On the other hand, Keap1 inhibitory protein was negatively correlated with MLSP in rodents, and it was almost not detected in liver of naked mole rats, whereas in laboratory mice Keap1 effectively halts Nrf2 activity [126]. The level of Nrf2-regulated genes, like those encoding HO-1, NQO1 or GSTA1, was also potently increased in naked mole rats in comparison to normal-living mice [126]. The summary of age-related changes in the Nrf2 regulatory system is shown in Fig. 6.

## Role of CncC/SKN-1/Nrf2 in proteasome regulation

**Fig. 7:**
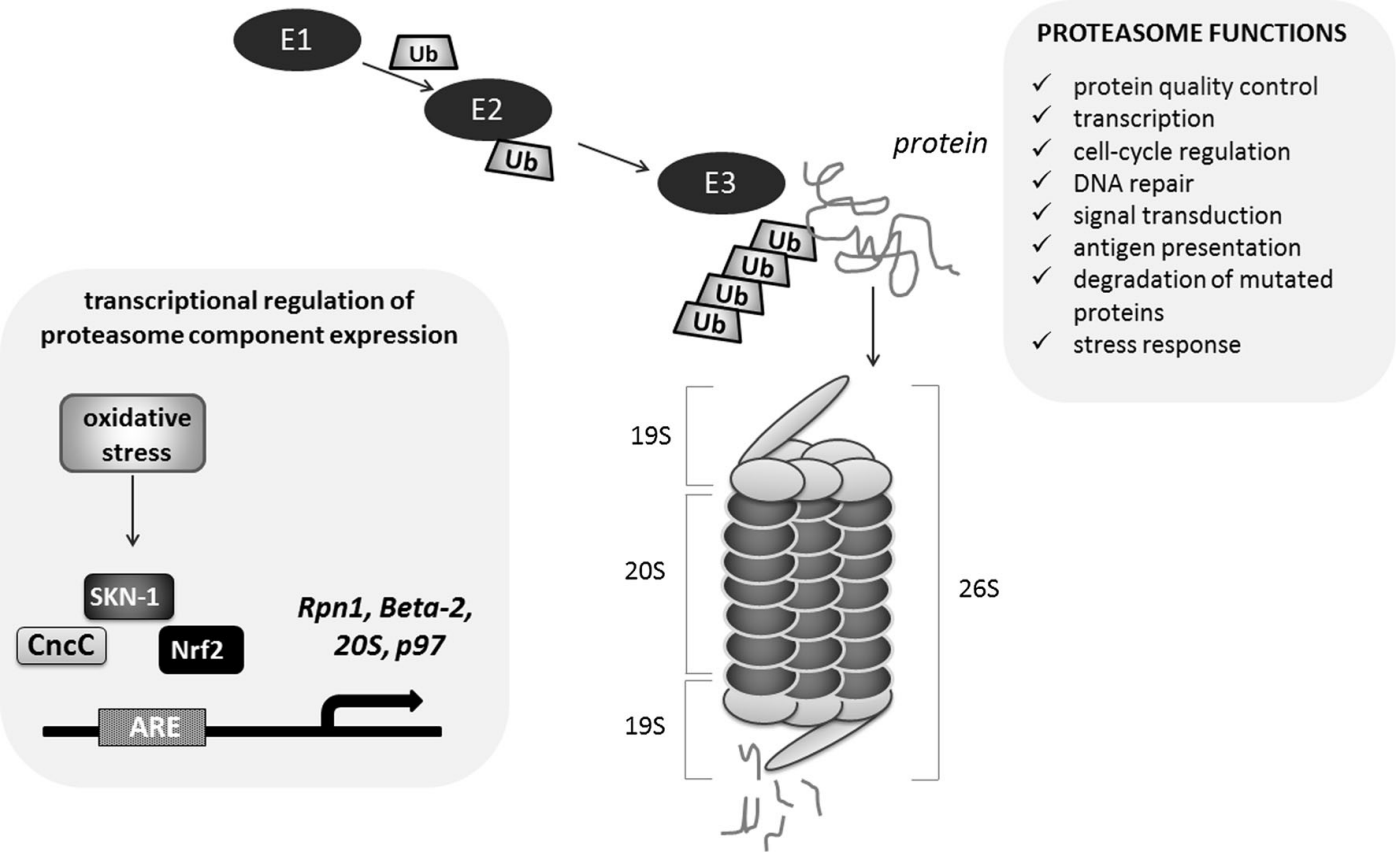
CncC/SKN-1/Nrf2 involvement in the regulation of proteasome. The Nrf2-dependent up-regulation of the proteasome levels, assembly, and activity may result in the adaptation to stressful conditions, lifespan extension and increased resistance to stress. The regulation of proteasome genes (*Rpn1, Beta*-*2, 20S, p97*) is indicated

During aging, a multifactorial process modulated by the interplay between genetic and environmental factors, the functionality of proteasomes declines in both the somatic tissues of *Drosophila* [127] and in mammalian cells [128]. The proteasome 26S is a complex of proteins composed of the proteolytic core (20S protein) flanked by regulatory particles, mostly ATPases (19S proteins), which acquire substrates and direct them into the proteolytic chamber. The 26S proteasome is mostly involved in the ATP-dependent degradation of normal short-lived ubiquitinated proteins, whereas the 20S proteasome itself degrades oxidized or damaged proteins [129]. A protein that is targeted for degradation is modified by E1, E2, E3 enzymes binding ubiquitin and a poly-ubiquitin chain is attached to the target protein. The 26S proteasome recognizes the poly-ubiquitin chain linked to the target protein and marks it as a substrate for hydrolysis (Fig. 7). This pathway is involved in the regulation of numerous cellular mechanisms, including protein quality control, transcription, cell-cycle regulation, DNA repair, signal transduction, antigen presentation and degradation of mutated proteins involved in the progression of many disorders, e.g., tumor development [130].

In *Drosophila*, CncC was identified as a candidate transcriptional regulator of proteasome component expression [131]. ARE-enhancer sequences, a target for CncC, are present in proteasome genes *Rpn1* and *Beta*-*2*, within location described as crucial for induction of proteasome gene transcription after treatment with an inhibitor in experimental conditions [132]. CncC is also involved in the increased 20S and p97 ATPase expression, but does not affect 26S. Similarly to mammals, *Drosophila* cells that are pre-treated with hydrogen peroxide adapt to oxidative stress by CncC-dependent up-regulation of the proteolytic activity and 20S proteasome expression [133]. Finally, it has been shown that age-dependent decrease in proteasome expression is related to CncC dysfunction in older flies [127, 134]. Tsakiri et al. showed that knockdown of 20S proteasome subunits causes lethality of larva [134]. The proteasome dysfunction observed in adults promotes “old-age” phenotypes and reduces the lifespan. These changes result from dysfunctions of CncC as *cncC* silencing reduced proteasome activity, stress resistance and longevity, while *cncC* up-regulation had the opposite effect. Interestingly, the prolonged *cncC* overexpression reduces longevity [134]. In another paper, Tsakiri et al., it was demonstrated that proteasome genes are differentially expressed in somatic and reproductive aged tissues and these changes are Cnc/Keap1-dependent [127]. Gonads of young flies had more active proteasomes than somatic tissues, and they were, independently of age, more resistant to oxidative stress. Additionally, the knockdown of *keap1* caused youthful proteasome gene expression level in aged somatic tissue, whereas *cncC* silencing resulted in the decreased proteasome gene expression in aged gonads, similar to that observed in normal aged somatic tissues. These findings explain the mechanism of “immortality” of reproductive cells, in which proteasome gene expression is high even in old animals [127].

The mechanism of enhanced proteasomal activity by Nrf2-induced gene expression represents the fundamental strategy in the protection against oxidative stress not only in flies, but also in nematodes and mammals. The worm orthologue of Nrf2, SKN-1 can promote longevity under reduced insulin/IGF-1-like signaling, dietary restriction, and normal conditions [116, 135].

SKN-1 and Nrf2 are involved in the adaptation to stressful conditions through the induction of the 20S proteasome [133]. In their recent study, Chondrogianni et al. demonstrated that up-regulation of the 20S proteasome levels, assembly, and activity results in *C. elegans* lifespan extension and increased resistance to stress [136]. Noteworthy, the extension of lifespan was dependent on the transcriptional activity of SKN-1 as well as the other factors like Dauer formation abnormal/Forkhead box class O (DAF-16/FOXO), and heat shock factor-1 (HSF-1) [136]. The involvement of DAF-16 (FOXO) in SKN-1 effects was also suggested by Wang et al. [135]. In mammalian cells, the activation of Nrf2 by sulforaphane (SFN) leads to increase of 20S proteasomal activity and to augmented expression of the 20S catalytic subunit gene, PSMB5 but this regulation is not present in the fibroblasts from *Nfe2l2*-disrupted mice [137]. Not only the 20S proteasome, but also increased level of the Pa28αβ (or 11S) proteasome regulator was reported to be regulated by Nrf2 as the adaptive response of the cells.

## Role of Nrf2/HO-1 in age-dependent diseases

As mentioned above, Nrf2 signaling pathway is essential for xenobiotic and oxidative stress responses and it plays a protective role in many different organs, and prevents kidney, liver, nervous system, lungs and other organs from the toxic insults. As oxidative stress is believed to contribute to aging, it could be easily predicted that Nrf2 system may function in the prevention of age-related diseases. Moreover, as discussed earlier, increasing proteasome activity might represent anti-senescence therapy and the defective ubiquitin/proteasome pathway leading to protein aggregates that is observed in many neurodegenerative disorders, suggesting a possible protective role of Nrf2/HO-1 system.

### Neurodegeneration

The basic research on the role of Nrf2 in astrocytes, neurons and other neuronal cells indicates the neuroprotective role of this transcription factor. Importantly, aging may affect Nrf2 level, and the reduction of Nrf2 mRNA and protein results in impaired Nrf2 signaling. Suh et al. [138] indicated that the level of Nrf2 and the expression of Nrf2-mediated γ-glutamylcysteine ligase (GCL) and glutathione (GSH) level in old (24–28 months) rats decrease potently in comparison to young (2–5 months) animals. In 2009, Duan et al. using in vitro and in vivo models, clearly showed that with aging, the level of Nrf2 as well as Nrf2-dependent genes, including HO-1, decreases in the mouse spinal cord and astrocytes [139]. Moreover, Suzuki et al. have found that Nrf2 level decreases in macrophages from older smokers in comparison with older nonsmokers. Simultaneously, the level of Keap1 and Bach1, the Nrf2 repressors increases in lungs [140].

The augmented oxidative stress is associated with death of cells of the nervous system in the pathogenesis of neurodegenerative diseases such as Alzheimer’s disease (AD), Parkinson’s disease (PD), Huntington’s disease (HD) or amyotrophic lateral sclerosis (Lou Gehrig’s disease). In vitro studies clearly indicate that chemical inducers of Nrf2, like tBHQ and SFN or its adenoviral overexpression in astrocytes results in protection against hydrogen peroxide-induced oxidative stress [141, 142]. On the other hand, experiments performed in Nrf2-deficient cells and Nrf2 knockout mice showed they are significantly more vulnerable to striatal damage reminiscent of HD [143]. A similar increase in the sensitivity of the Nrf2-deficient mice has been observed in models of PD. The neurotoxin 6-hydroxydopamine (6-OHDA) both in vitro and in vivo showed a greater effect in Nrf2-deficient cells than in wild-type counterparts [144]. Similarly, in the 1-methyl-4-phenyl-1,2,3,6-tetrahydropyridine (MPTP) animal model of PD, the greater loss of dopamine transporter levels in the striatum of Nrf2 knockout mice than in wild-type at all MPTP doses tested was observed [145, 146]. Moreover, Nrf2 activation by SFN protects against MPTP-induced death of nigral dopaminergic neurons, leading to decrease in astrogliosis, microgliosis, and release of pro-inflammatory cytokines [147]. Interestingly, HO-1^−/−^ mice have been shown to be similarly sensitive to MPTP as wild-type littermates [146], although the previous studies showed ample evidence for neuroprotective activities of HO-1, e.g., adenoviral overexpression of HO-1 increased significantly the survival rate of dopaminergic neurons in the rat model of PD [148]. Of note, as mentioned above, some studies indicate that carriers of -413 A/T SNP in HO-1 together with TT genotype of GSK3β had four times higher risk of developing PD than subjects without these genotypes [89].

In flies, CncC can also protect from neurodegeneration, as it has been shown in the *Drosophila* model of PD. The chemical agents, SFN and allyl disulfide, inhibit neurodegenerative changes by induction of CncC activity [149]. It has also been shown that decaffeinated coffee and nicotine-free tobacco (but not caffeine and nicotine alone) have neuroprotective effect on flies with overexpression of human α-synuclein or in parkin mutant (models of PD), as well as in the fly’s models of AD and polyglutamine disease. This effect is mediated by Cnc, as it has been proved by silencing *cnc* transcripts that suppressed the cytoprotective effect of coffee and tobacco. Moreover, the Cnc activator present in coffee, called cafestol, is able to protect neuronal cells against death in transgenic α-synuclein flies. Cafestol can induce gstD1-GFP that is used as a reporter of Nrf2 activity [150]. Barone et al. claim that the Cnc overexpression in dopaminergic neurons is sufficient to counteract α-synuclein toxicity [151]. In the recent paper by Wang et al., transgenic flies with mutated α-synuclein (A53T), showing neurodegenerative changes characteristic for PD, were protected by bardoxolone methyl (CDDO-Me), Nrf2 inducer, through ROS scavenging and the activation of Nrf2/antioxidant response element signaling pathway. Furthermore, the selective expression of CncC in dopaminergic neurons effectively protected against the neurodegenerative phenotype of the A53T α-synuclein flies [152].

The involvement of Nrf2 in the AD development seems to be associated with the disease progression. Several studies have shown the increase in the expression levels of Nrf2-target genes, including HO-1 during AD progression [153, 154]. The another studies showed a potent up-regulation of HO-1 in glial cells of AD patients [155]. In addition, the inhibition of HO-1 by azole-based, a brain-permeable inhibitor of HO-1, OB-28, led to the significant improvement in a complex maze learning task relative to saline-treated controls, indicating the amelioration of behavioral anomalies in the transgenic mouse model of AD through HO-1 down-regulation [156]. However, in 2007, Ramsey and colleagues have observed the decrease in nuclear Nrf2 expression in the affected brain regions of AD patients [157]. As it was suggested by Yamazaki et al., in the early stages of AD, Nrf2 is up-regulated by β-amyloid-induced ROS, whereas during the disease progression Nrf2 level starts to decrease [158].

The neuroprotective role of Nrf2 is based also on the results from dimethyl fumarate (DMF) action in multiple sclerosis (MS). MS is a chronic, devastating, autoimmune disease manifested by the demyelination of neuronal axons. Inflammation and oxidative stress together with neurodegeneration contribute to disease progression (reviewed in [159]).

The protective role of DMF in MS pathology is supported by both in vitro and in vivo studies as well as by the results of large clinical trials (CONFIRM and DEFINE) [160]. Based on those experiments it was suggested that DMF does not have a single mechanism of action, but rather exerts a multitude of biological effects. It possess anti-inflammatory potential through shifting the cytokine profile from Th1 toward a Th2 profile as well as it was shown to inhibit the activation of NF-κB and pro-inflammatory IL-6 and IL-12 (reviewed in [161, 162]). Additionally, in 2011, Linker et al. demonstrated that beneficial effects of DMF in murine experimental autoimmune encephalomyelitis (EAE), a mouse model of MS are mainly due to activation of Nrf2 [163]. Many other papers showed that the regulation of Keap1 and Nrf2 activation, resulting in transcription of HO-1 or NQO1 is responsible for neuroprotective effect of DMF (reviewed in [161]).

Importantly, the DMF analog, BG-12 (brand name Tecfidera™) was approved in 2013 for the treatment of relapsing-remitting MS by the European Union and the US Food and Drug Administration [160]. Not only MS patients may be treated by DMF, as it reduces psoriasis symptoms [164] as well it might protect against systemic and CNS complications in HIV infection through its effective suppression of immune activation, oxidative stress, HIV replication, and macrophage-associated neuronal injury [165].

**Fig. 8:**
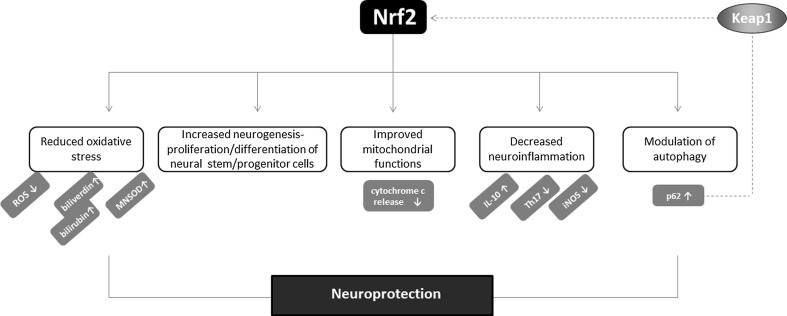
Neuroprotective activity of Nrf2. Nrf2 mediates neuroprotection through several possible mechanisms. The reduction of oxidative stress, modulation of inflammation as well as autophagy process may contribute to protective effects of Nrf2 in the nervous system. Of note, p62 may directly activate Nrf2 through sequestration of Keap1

Several possible mechanisms could be responsible for the neuroprotective activity of Nrf2 (Fig. 8). First of all, the induction of protective genes, like those encoding HO-1 and GCL or GSH synthetase may be a major component of the protection against oxidative stress exerted by Nrf2 [142]. Nrf2 is also crucial for injury-induced neurogenesis in the hippocampus, regulating proliferation and neuronal differentiation of neural stem/progenitor cells [166]. Possibly, Nrf2 may protect from mitochondrial dysfunction and prevent from increased ROS generation and death of neural cells. In addition, the control of neuroinflammation by Nrf2, and the inhibition of pro-inflammatory factors, like IL-6 or iNOS as well as the up-regulation of anti-inflammatory IL-10 may contribute to this effect. Moreover, Nrf2 may modulate autophagy since defective autophagic system is known to be pathogenic in neurodegenerative disorders like AD [167].

As mentioned above, under normal conditions, Nrf2 is bound to Keap1 and is inactivated as a transcription factor for antioxidant defense genes by proteasome-mediated degradation. Under oxidative stress, Keap1 can be modified through oxidative-dependent mechanism and it can no longer bind Nrf2. It might also be sequestered by p62, a ubiquitin-binding protein acting as a scaffold for several protein aggregates and triggering their degradation through the proteasome, or the lysosome pathway via autophagy [168, 169]. In 2010, the non-canonical pathway of Nrf2 activation, relying on redox-independent interaction between Keap1 and p62 was suggested [168]. Lau et al. have shown that accumulation of endogenous p62 or ectopic expression of p62 sequester Keap1 into aggregates, resulting in the inhibition of Keap1-mediated Nrf2 ubiquitination and its subsequent degradation by the proteasome [168]. Moreover, phosphorylation of p62 at Ser^351^ increases its binding affinity for Keap1, leading to the increased expression of cytoprotective Nrf2 targets [170]. Jain et al. identified p62 as a direct Nrf2-regulated gene as Nrf2 binds to ARE sequence in the p62 promoter, leading to its increased expression [171]. In turn, in genetically modified mice with decreased 26S proteasome activity, the activation of autophagy and the Keap1-Nrf2 pathway represent the defense mechanisms that is able to adapt to impaired proteasome function [172].

### Age-related macular degeneration

**Fig. 9:**
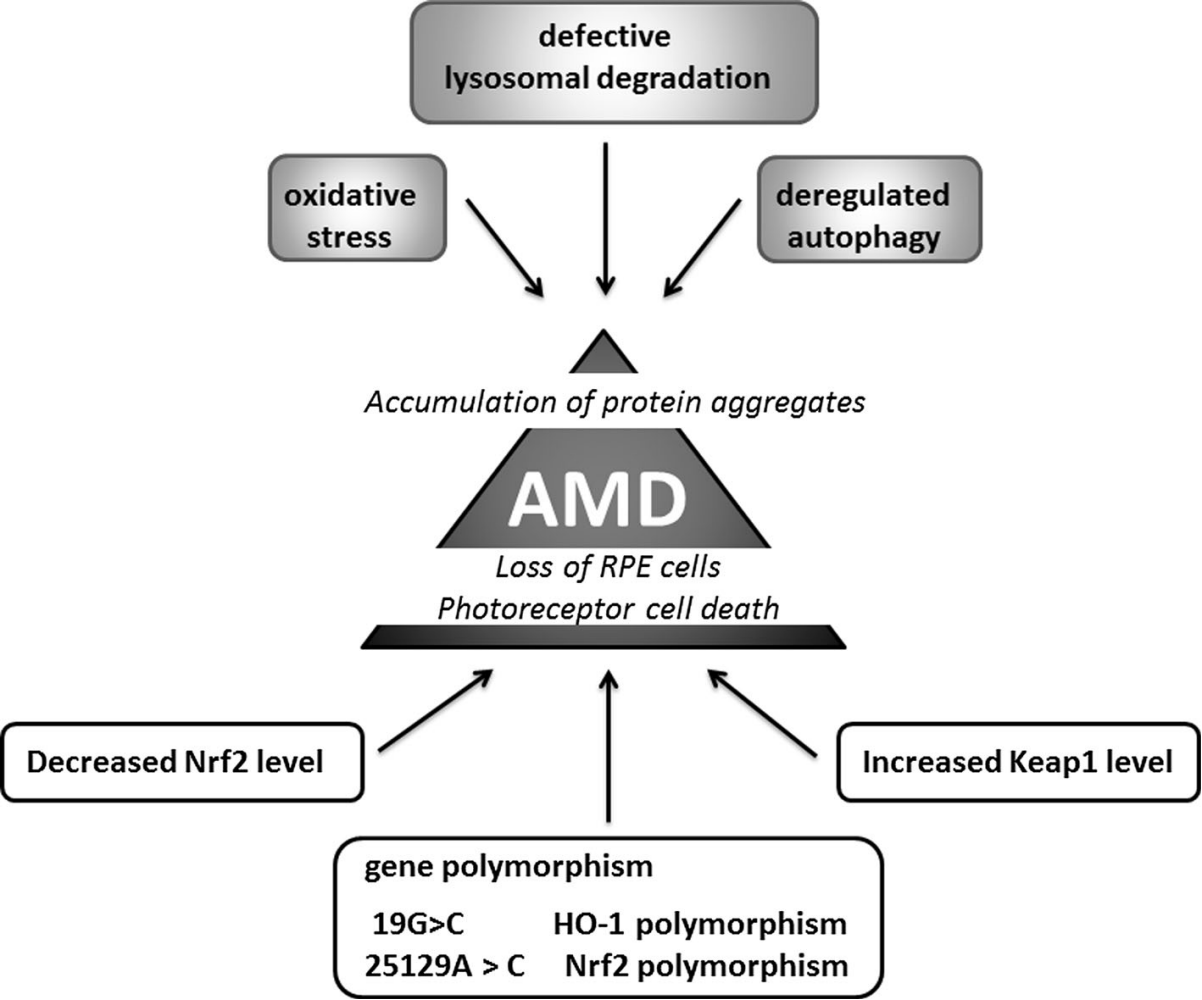
Age-related macular degeneration and Nrf2. Decrease in Nrf2 and increase in Keap1 levels as well as Nrf2 and HO-1 promoter polymorphism contribute to damage of the retinal pigment epithelium (RPE) cells and photoreceptors. Nrf2 deficiency leads to increased oxidative stress and deregulated autophagy magnified the accumulation of protein aggregates and drusen formation

Oxidative stress might be also one of the main factors contributing to the pathogenesis of age-related macular degeneration (AMD), characterized by the damage of the retinal pigment epithelium (RPE) cells and photoreceptors (Fig. 9). In aging Nrf2 KO mice degeneration of RPE cells resembling human AMD is evident with defects in lysozyme-dependent degradation leading to less efficient removal of oxidatively damaged protein aggregates through deregulated autophagy [173]. Interestingly, the 25129A>C polymorphism of the Nrf2 gene has been suggested to be associated with AMD [174]. Similarly, the genetic variation in HO-1 gene may affect the pathogenesis of AMD through the modulation of the cellular reaction to oxidative stress. It has been suggested that 19G>C-HO-1 polymorphisms may be associated with the occurrence and progression of AMD [175].

The possible link between Nrf2, AMD and cigarette smoking, the latter known to be involved in the pathology of AMD [176], has been studied by Wang et al. [177]. Indeed, Nrf2 deficiency magnified the effect of cigarette smoking on inflammatory response and complement activation, the effects contributing to drusen formation [177]. On the other hand, in vitro experiments performed on ARPE-19 cells exposed to cigarette smoke extract (CSE) or hydroquinone (HQ), a component of cigarette smoke, showed that the increased oxidative damage and apoptosis, is manifested by HO-1 up-regulation and possibly iron accumulation [178]. Given the evidence that iron accumulation within the eye may account for pathological progression of AMD [179], the very high activity of HO-1 caused by CSE may affect AMD pathology.

The direct comparison of young (2 months) and old (15 months) mice under unstressed and sodium iodate-stressed conditions, that is the frequently used model for the retinal degeneration [180], have underlined the involvement of Nrf2 signaling in the pathogenesis of the disease [181]. The retinal pigment epithelium of old mice exhibited impaired induction of Nrf2-dependent genes in response to the toxic insult. On the other hand, genetic enhancement of Nrf2 activity by the conditional knockdown of the Nrf2 negative regulator Keap1, partially restores Nrf2 signaling through the increased NQO1 expression in old mice [181].

### Cancer

Growing body of evidence suggests that Nrf2 is involved in the chemoprevention of normal cells but also promotes the growth of cancer cells. Similarly, HO-1 involvement in cancer progression is well documented, but also HO-1 may be protective for cancer cells at least in some tumor types.

Noteworthy, ROS are involved in both tumor initiation as well as in tumor progression, causing nucleic acid alterations, both sugar and base modifications in nuclear but also in mitochondrial DNA that is more prone to mutations due to the lack of histones [182]. In tumors, the production of ROS is exacerbated through the increased mitochondrial activity and accelerated metabolism of tumor cells. ROS scavenging through the Nrf2-mediated induction of target genes might be a protective mechanism of action of many natural and synthetic compounds used as chemopreventive agents, which are able to stop or delay the occurrence of malignancy (Fig. 7). The examples include oltipraz, oleanolic acid (OA) and its synthetic derivatives, like CDDO-imidazole (CDDO-Im) (1-[2-cyano-3-,12-dioxooleana-1,9(11)-dien-28-oyl] imidazole) or SFN (reviewed in [183, 184]). In our hands, some synthetic derivatives of OA increased Nrf2 activity and its target gene expression, but we also observed cell-type specific response to other triterpenoid, betulin on Nrf2 activation in keratinocytes and endothelial cells [183]. Additionally, the beneficial, Nrf2-dependent effects have been clearly presented in many in vitro and in vivo studies also using Nrf2-deficient cells/animals. The effectiveness of Nrf2 inducers has been assessed in clinical trials. However, oltipraz as well as the CDDO-derivative, bardoxolone methyl, because of serious side effects reported in some trials, like BEACON—bardoxolone methyl evaluation in patients with chronic kidney disease and type 2 diabetes (ClinicalTrials.gov, NCT01351675), have been withdrawn from clinics. The plethora of data from Nrf2 KO mice suggest greater susceptibility of such mice to carcinogenic insults, like *N*-nitrosobutyl(4-hydroxybutyl)amine (BBN)-induced bladder carcinogenesis [185], aflatoxin-induced human hepatocellular carcinomas (HCCs) [186] or ultraviolet-triggered skin carcinogenesis [187].

Although the positive, protective effect of Nrf2 has been reported mostly during tumor initiation, it is now well documented that this transcription factor has a dual role in tumorigenesis. In the last years, an increasing number of studies have examined the oncogenic properties of Nrf2 (reviewed in [188]) as shown in lung cancer [189], squamous cell carcinomas of esophagus and skin [190] or hereditary leiomyomatosis and renal cell cancer (HLRCC) [191]. HLRCC is caused by a mutation of fumarate hydratase (FH), the Krebs cycle enzyme, functioning as a tumor suppressor gene and the inactivation of FH initiates formation of multiple renal cysts. Adam et al. showed that the formation of cysts in FH-deficient mice is dependent on Nrf2 and on the modification of Keap1 [191]. In lung cancer, loss of Keap1 function leading to constitutive activation of Nrf2-mediated gene expression caused enhanced transcriptional induction of antioxidants, xenobiotic metabolism enzymes, and drug efflux pumps and promotes tumorigenesis [189]. Of note, mutation of Keap1 leading to its inactivation is a frequent genetic alteration in non-small-cell lung carcinoma (NSCLC) [189].

Similarly, HO-1 demonstrated pro-tumorigenic effects in many cancers. HO-1 was shown to increase tumor cells proliferation and migration and prevent cancer cells from apoptosis and autophagy (for review see [192]). The regulation of blood vessel formation and the increase in the expression of pro-angiogenic factors are also regulated in HO-1 dependent way ([193–195], reviewed in [67, 196]). The positive correlation between the progression of tumors and increased HO-1 expression was noted for many tumors, including prostate cancer, renal cancer, glioma, melanoma, pancreatic cancer, Kaposi sarcoma and others (for review see [196]).

Recent studies underlined the possible link between oncogenic mutations and HO-1 induction. *Hmox*-*1* gene expression is induced upon expression of oncogenes associated with the induction of some types of cancer. In Kaposi sarcoma, HO-1 mediates the oncogenic G protein-coupled receptor (vGPCR) oncogene-induced transformation associated with the increased vascular endothelial growth factor (VEGF) synthesis [197, 198]. The other study indicates the link between HO-1 and Bcr/Abl fusion gene in chronic myelogenous leukemia (CML) [199], whereas in kidney cancer the activation of Ras pathway and the stimulation of downstream signaling, including Raf and ERK, lead to Nrf2 activation and HO-1 induction [200]. In addition, *Drosophila* was used as a model for studying link between Myc, a pro-oncogenic transcription factor which has been found to be deregulated in a large number of different cancer types, and Nrf2. Nagy et al. have reported that the activation of Cnc/Nrf2 is required for Myc-induced overgrowth and this effect is related to the autophagy unfolded protein response induction, p62 accumulation and Nrf2 activation [201]. In 2010 it was already published, that p62 activation, through the constant activation of antioxidant response via the disruption of the interaction between Keap1 and Nrf2, leads to tumorigenesis [169]. The Myc-induced overgrowth in *Drosophila* is a consequence of both increased autophagy and antioxidant response. In mammals, the direct interaction of c-Myc and Nrf2 and c-Myc-dependent negative regulation of phase II genes was demonstrated [202]. The cross-talk between Nrf2 and other transcription factors playing an important role in cancer cell biology has to be also underlined. We have demonstrated that the regulation of pro-angiogenic IL-8 expression by HIF-1 and HIF-2 transcription factors requires Nrf2 as well as c-Myc factors [203].

**Fig. 10:**
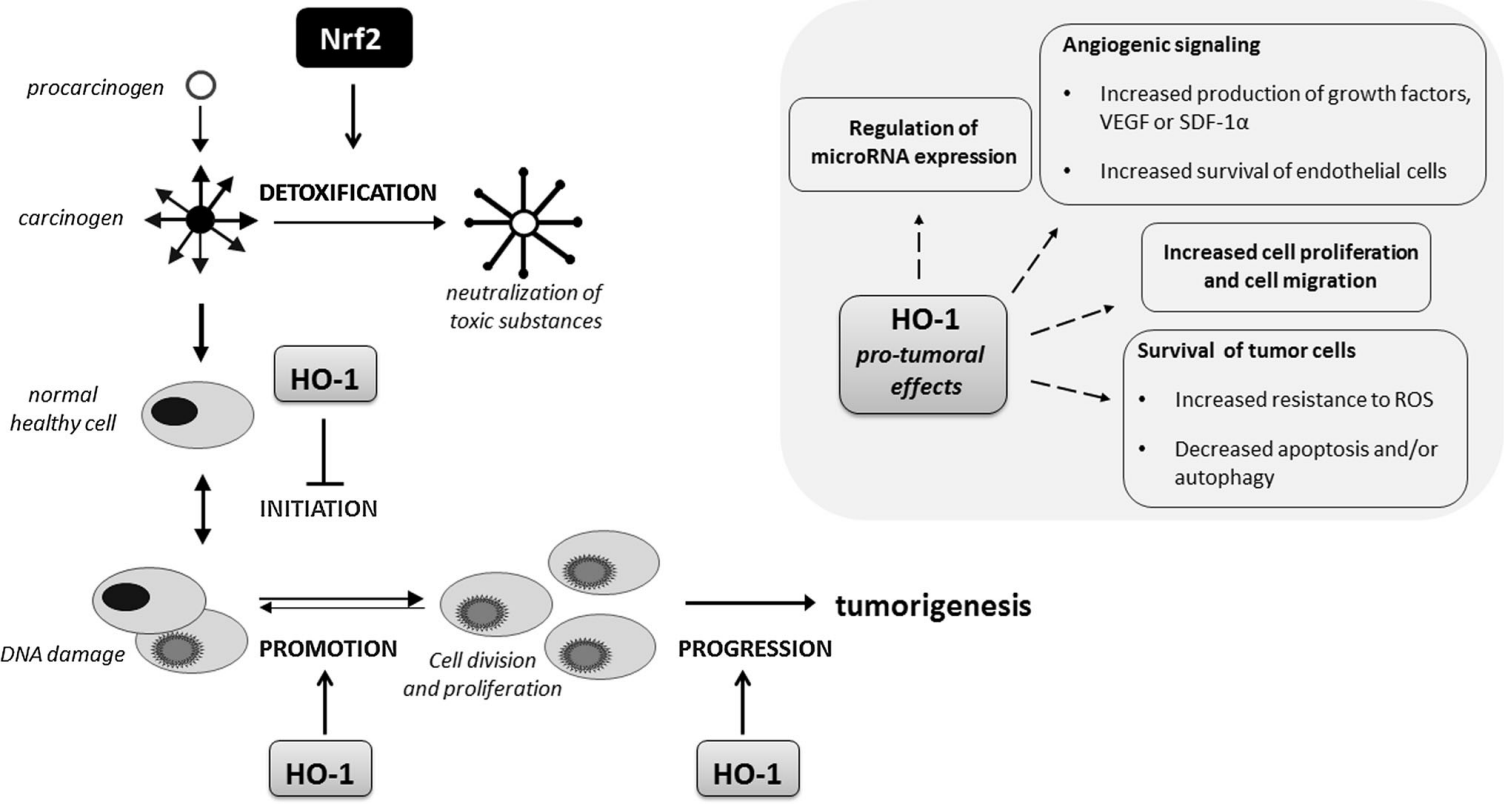
Role of Nrf2/HO-1 in tumor initiation and progression. Both Nrf2 and HO-1 may decrease tumor initiation through the detoxification and ROS scavenging mechanisms. On the other hand, this cytoprotective system exerts stimulatory effect on the progression of tumors mostly due to its anti-apoptotic, pro-angiogenic, pro-migratory and modulatory influence on the expression of microRNAs. However, in some tumors the opposite, inhibitory effect on progression of tumors have been reported

Not only pro-tumoral effects of HO-1 are described; due to its antioxidant and genome protecting activities, HO-1 may exert the protective effects against carcinogens and may reduce the probability of tumor initiation (Fig. 10). In agreement with that, we have observed that HO-1 may protect healthy tissues against chemical induction of squamous cell carcinoma, but in already growing tumors HO-1 accelerates its progression toward more malignant forms [204]. The dual role of HO-1 and its products, mostly CO, in tumorigenesis depending on tumor type and the balance between anti-angiogenic and proangiogenic pathways activated in tumors by HO-1 was described in several review papers including our recent review [70] and summarized also in Fig. 10.

## Nrf2 as a factor contributing to reductive stress

As mentioned above, Nrf2 is protective gene; however, recent studies underline the fact that its constant overexpression might be detrimental. Wakabayashi et al. observed that deletion of Keap1 leads to Nrf2 accumulation in nucleus at constitutively high levels and the overproduction of cytoprotective enzymes. Unexpectedly, Nrf2 accumulation was not protective, but postnatal lethality was observed as the result of Keap1 deletion [205]. Recent studies focused at the understanding of this phenomenon, and the involvement of Nrf2 in so-called reductive stress was suggested. Reductive stress is induced by conditions which promote the formation of excessive intracellular NAD(P)H together with enormous activation of antioxidant system and suppressed oxidative activity (reviewed in [206]). The increased level of GSH, Nrf2 target gene, might also act as a reducing equivalent, similar to NAD(P)H. In fact, it was shown that the deleterious effects of reductive stress were associated with dysregulation of glutathione homeostasis (increased GSH level and ratio of GSH/GSSG) and protein aggregation cardiomyopathy in experimental mice model [207, 208]. Further experiments identified Nrf2 as a factor contributing to reductive stress in the human mutant protein aggregation cardiomyopathy (MPAC) in mice [209]. Moreover, using MPAC transgenic mice, Kannan et al. evidenced that attenuation of Nrf2 signaling prevents from reductive stress. Genetic deletion of Nrf2 in such transgenic MPAC animals reduces aggregation of toxic mutant proteins and prevents pathological cardiac remodeling caused by reductive stress [210]. The possible involvement and underlying mechanisms of Nrf2-dependent reductive stress is summarized in recent review by Narasimhan and Rajasekaran [206].

In summary, the maintaining Nrf2-Keap1 activity at optimal levels to deal with both oxidative and reductive stress and maintaining proper redox homeostasis is crucial for the proper cell functioning.

## MicroRNAs and Nrf2/HO-1 system

**Fig. 11:**
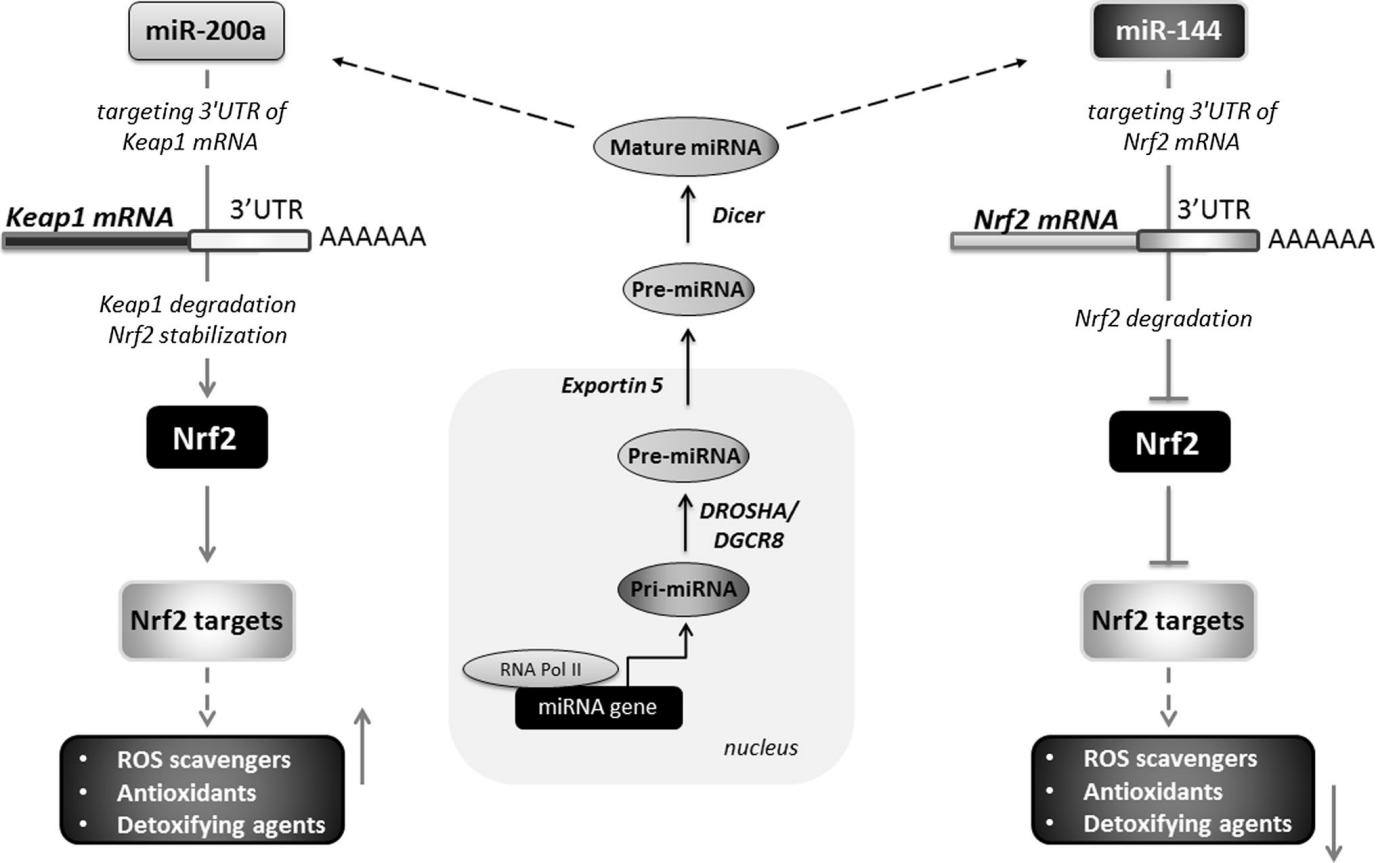
miRNAs biogenesis and their role in the regulation of Nrf2 expression. microRNAs, transcribed by polymerase II as pri-miRNAs, are cleaved by endoribonucleases Drosha and DGCR8, to be finally exported from the nucleus by exportin-5. An endoribonuclease Dicer processes the pre-miRNA, creating a mature, double-stranded miRNA duplex. Endogenous miRNAs bind to target sequences in the 3′UTR regions of their target mRNA to produce translational arrest. Some miRNAs, e.g., miR-200a can target Keap1 mRNA, leading to its degradation and up-regulation of Nrf2 targets. On the other hand, miR-144 can affect Nrf2 level in a Keap1-independent manner but through direct targeting the 3′UTR of Nrf2 mRNA causing the down-regulation in Nrf2 expression

As microRNAs are the master regulators of gene expression and their level is frequently altered in age-related disorders like cancer or neurodegenerative diseases, it could be easily predicted that these small regulatory RNA molecules serve important functions in modulating the Nrf2/HO-1 signaling pathway (Fig. 11). However, the role of microRNAs in the regulation of Nrf2 expression has been studied in limited number of publications. Chorley et al. using ChIP-seq analysis identified several microRNAs which may potentially regulate Nrf2 activity [211]. The direct evidence of Nrf2 regulation by the specific microRNAs has been shown by Eades et al. who found that miR-200a that targets Keap1 is a regulator of Nrf2 activation in breast cancer cells [212]. On the other hand, miR-28 regulates Nrf2 in a Keap-1-independent way by targeting the 3′UTR of Nrf2 mRNA and decreases Nrf2 expression in mammary epithelial cells [213]. Another microRNA shown to inhibit Nrf2 mRNA expression through direct effect on 3′UTR in Nrf2 mRNA was miR-144 [214].

Nrf2-dependent regulation of the microRNAs important for some aspects of tumorigenesis has been identified. For example, Nrf2 abrogates mir-1 and miR-206 and affects lung carcinoma metabolism by reprogramming glucose metabolism toward the pentose phosphate pathway (PPP) and the tricarboxylic acid (TCA) cycle [215]. Similarly, we showed the inhibitory effect of HO-1 on myomirs, including miR-206 expression in C2C12 cells, and myoblast differentiation [216]. Moreover, we have also demonstrated that HO-1 inhibits miR-378 and the interplay between HO-1 and miR-378 significantly modulates NSCLC progression and angiogenesis [217, 218]. Interestingly, although HO-1 enhanced expression of microRNAs processing enzymes (DGCR8, Drosha and Dicer1) in NCI-H292 cell line and increased global amounts of miRNA fraction [217], the expression of some specific microRNAs was inhibited. For example, not only miR-378, but also the expression of other pro-angiogenic microRNAs, such as miR-17-92 and miR-210 were downregulated. On the other hand, anti-angiogenic miR-424 was upregulated by HO-1. Consistently tumors from HO-1 overexpressing NCI-H292 cells were less vascularized and grew slower [217].

As mentioned above, Nrf2/HO-1 system plays a pivotal role in neurodegenerative disorders, and a link with microRNAs was found also in this subject. In primary astrocytes overexpressing HO-1, the up-regulation of miR-16, miR-17 and miR-140 with concomitant inhibition of miR-29c, miR-138, miR-181a, miR-187, miR-206 and miR-297 was observed [219]. The changes in the expression of those microRNAs and regulated mRNAs might be responsible for the inflammatory, degenerative and apoptosis-triggered cell death.

All these results concerning the microRNAs regulation further clarify molecular mechanisms by which Nrf2/HO-1 participate in pathogenesis and therapy of stress activated and age-related diseases.

## Conclusions

Nrf2/HO-1 pathways are widely studied in vertebrates. As SKN-1 in *C. elegans* or CncC in *D. melanogaster* share high homology with Nrf2, these organisms offer suitable models to study the function and biology of Nrf2 transcription factors. A large number of experiments indicate the importance of Nrf2 and its equivalents in embryonic development, stress signaling and aging. Moreover, Nrf2-dependent genes, like HO-1 provide cytoprotective effect and play a crucial role in the development of oxidative and age-related disorders. The more detailed analysis of microRNAs involvement in the regulation of Nrf2/HO-1 may provide new ideas for the treatment of abovementioned diseases. The involvement of Nrf2 in the reductive stress in the mouse model of protein aggregation cardiomyopathy suggests the contribution of this transcriptional regulator of antioxidant genes in other pathological situations related to unbalanced redox homeostasis. Although our knowledge about the role of Nrf2 in controlling both physiology and disease progression extended rapidly recently, thanks to model organisms including flies and worms, still the influence of CNC factors on lifespan and health is an attractive scientific problem to resolve.
